# Very Low‐Intensity Ultrasound Facilitates Glymphatic Influx and Clearance via Modulation of the TRPV4‐AQP4 Pathway

**DOI:** 10.1002/advs.202401039

**Published:** 2024-11-04

**Authors:** Chueh‐Hung Wu, Wei‐Hao Liao, Ya‐Cherng Chu, Ming‐Yen Hsiao, Yi Kung, Jaw‐Lin Wang, Wen‐Shiang Chen

**Affiliations:** ^1^ Department of Physical Medicine and Rehabilitation National Taiwan University Hospital and National Taiwan University College of Medicine Taipei 100 Taiwan; ^2^ Department of Physical Medicine and Rehabilitation National Taiwan University Hospital Hsin‐Chu Branch Hsinchu 300 Taiwan; ^3^ Department of Biomedical Engineering College of Medicine and College of Engineering National Taiwan University Taipei 106 Taiwan; ^4^ Department of Biomechatronic Engineering National Chiayi University Chiayi 600 Taiwan; ^5^ Institute of Biomedical Engineering and Nanomedicine National Health Research Institutes Miaoli 350 Taiwan

**Keywords:** aquaporin‐4, glymphatic, transient receptor potential vanilloid‐4, ultrasound, β‐amyloid

## Abstract

Recently, the glymphatic system has been proposed as a mechanism for waste clearance from the brain parenchyma. Glymphatic dysfunction has previously been shown to be associated with several neurological diseases, including Alzheimer's disease, traumatic brain injury, and stroke. As such, it may serve as an important target for therapeutic interventions. In the present study, very low‐intensity ultrasound (VLIUS) (center frequency, 1 MHz; pulse repetition frequency, 1 kHz; duty factor, 1%; spatial peak temporal average intensity [I_spta_] = 3.68 mW cm^2^; and duration, 5 min) is found to significantly enhance the influx of cerebrospinal fluid tracers into the paravascular spaces of the brain, and further facilitate interstitial substance clearance from the brain parenchyma, including exogenous β‐amyloid. Notably, no evidence of brain damage is observed following VLIUS stimulation. VLIUS may enhance glymphatic influx via the transient receptor potential vanilloid‐4‐aquaporin‐4 pathway in astrocytes. This mechanism may provide insights into VLIUS‐regulated glymphatic function that modifies the natural course of central nervous system disorders related to waste clearance dysfunction.

## Graphical abstract

The study investigates the impact of very low‐intensity ultrasound (VLIUS) on glymphatic function, revealing its potential to enhance cerebrospinal fluid influx and β‐amyloid clearance in the brain through the TRPV4‐AQP4 pathway. The findings suggest that VLIUS can be a promising therapeutic approach for neurological disorders associated with glymphatic dysfunction, such as Alzheimer's disease and traumatic brain injury.

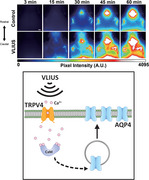

## 1 Introduction

Waste clearance is important for maintaining the normal functioning of the central nervous system (CNS). The glymphatic system has recently been identified as an important mechanism which functions to clear waste from the brain parenchyma. The current model of the glymphatic system proposes an influx of cerebrospinal fluid (CSF) from the subarachnoid space into the brain parenchyma through the periarterial spaces, mixing with parenchymal interstitial fluid (ISF) and waste products facilitated by aquaporin‐4 (AQP4) channels in the endfeet of astrocytes, resulting in drainage through the perivenous spaces.^[^ ^1^ ^]^ Glymphatic dysfunction has previously been associated with several neurological diseases in animal models, such as Alzheimer's disease,^[^ ^2^ ^]^ traumatic brain injury,^[^ ^1^, ^3^ ^]^ stroke,^[^ ^4^ ^]^ migraine,^[^ ^5^ ^]^ multiple sclerosis,^[^ ^6^ ^]^ and amyotrophic lateral sclerosis.^[^ ^7^ ^]^ Because of its pathophysiological association with a broad range of CNS diseases, the glymphatic system has been considered a potentially important target for therapeutic intervention.

Interactions between the glymphatic system and various intrinsic and extrinsic factors, such as sleep, body posture, blood pressure, aging, and anesthesia have previously been reported. Further, research has shown that the glymphatic influx decreases following sleep deprivation,^[^ ^8^ ^]^ and was more effective in the right lateral decubitus position than in the prone position.^[^ ^9^ ^]^ Epinephrine‐induced acute hypertension considerably reduced the influx of CSF tracers.^[^ ^10^ ^]^ Decreased glymphatic clearance has also been observed in older brains.^[^ ^11^ ^]^ Some anesthetics (e.g., xylazine and dexmedetomidine) show higher CSF tracer influx, similar to that observed during spontaneous sleep, whereas others (e.g., pentobarbital and isoflurane) significantly inhibit glymphatic influx.^[^ ^12^ ^]^ As the glymphatic system aids in the removal of waste, such as β‐amyloid and tau proteins, from the CNS, and can be modulated by various factors, exploring ways to enhance glymphatic function may be a good therapeutic approach for the treatment of CNS disorders related to waste clearance dysfunction.

AQP4 is characterized by its paravascular distribution, and is closely associated with glymphatic function.^[^ ^1a^ ^]^ Studies on AQP4‐knockout mice revealed uninterrupted influx within the periarterial spaces; however, the flow of tracers from these spaces to the surrounding parenchyma was significantly hindered, indicating that the role of AQP4 in facilitating fluid movement between the paravascular and interstitial spaces.^[^ ^1a^ ^]^ Notably, Snta1‐knockout mice exhibited regular AQP4 expression, but manifested a deficiency in AQP4 polarization, which consequently leads to diminished glymphatic flow, similar to AQP4‐knockout mice.^[^ ^13^ ^]^ This observation underscores the importance of AQP4 polarization in the maintenance of proper glymphatic function. Calmodulin (CaM) is directly associated with the carboxyl terminus of AQP4, triggering a distinct conformational alteration that drives AQP4 polarization; importantly, the polarization process of AQP4 is susceptible to inhibition by trifluoperazine, a CaM inhibitor.^[^ ^14^ ^]^ One other study employing coimmunoprecipitation and immunohistochemistry analyses demonstrated interaction and co‐localization of AQP4 with transient receptor potential vanilloid 4 (TRPV4),^[^ ^15^ ^]^ notably highlighting the presence of a TRPV4/AQP4 complex, which plays a critical role in maintaining brain volume homeostasis, within astrocytes. Hence, the complex interplay between TRPV4, CaM, and AQP4 may underscore the potential of their modulation to intricately influence glymphatic function.

Mechanical stimulation may also affect glymphatic function. For example, one study showed that fluid shear stress, analogous to that produced by paravascular CSF or ISF dynamics, could mechanically stimulate N‐methyl‐D‐aspartate receptors on astrocytes, producing increased calcium ion (Ca^2+^) currents and indicating a role of mechanotransduction in glymphatic flow.^[^ ^16^ ^]^ Another study demonstrated the crucial role of TRPV4 in ultrasound (without microbubbles)‐mediated blood‐brain barrier (BBB) permeability.^[^ ^17^ ^]^ Transcranial ultrasound without microbubbles enhances the influx of cerebrospinal fluid into the paravascular spaces of the brain, glymphatic system, and brain parenchyma.^[^ ^18^ ^]^ Moreover, one recent study indicated that focused ultrasound with microbubbles could enhance the glymphatic–lymphatic clearance of β‐amyloid, predominantly by increasing brain‐to‐CSF β‐amyloid drainage.^[^ ^19^ ^]^ Based on these findings, we hypothesized that ultrasound may be a promising therapeutic intervention for degenerative CNS disorders. Furthermore, we propose that one of the underlying mechanisms by which ultrasound exerts its beneficial effects is the enhancement of glymphatic function via the TRPV4/AQP4 pathway.

In the present study, we investigated the changes in glymphatic dynamics induced by ultrasound. Our previous study revealed a significant capacity of very low‐intensity ultrasound (VLIUS) to stimulate neurogenesis in specific regions of the mouse brain without detrimental effects.^[^ ^20^ ^]^ In the present study, we aimed to elucidate the connections between VLIUS, TRPV4, and AQP4, with a focus on their roles in modulating glymphatic function.

## 2 Results

### 2.1 VLIUS Increased CSF Tracer Influx

**Figure 1:**
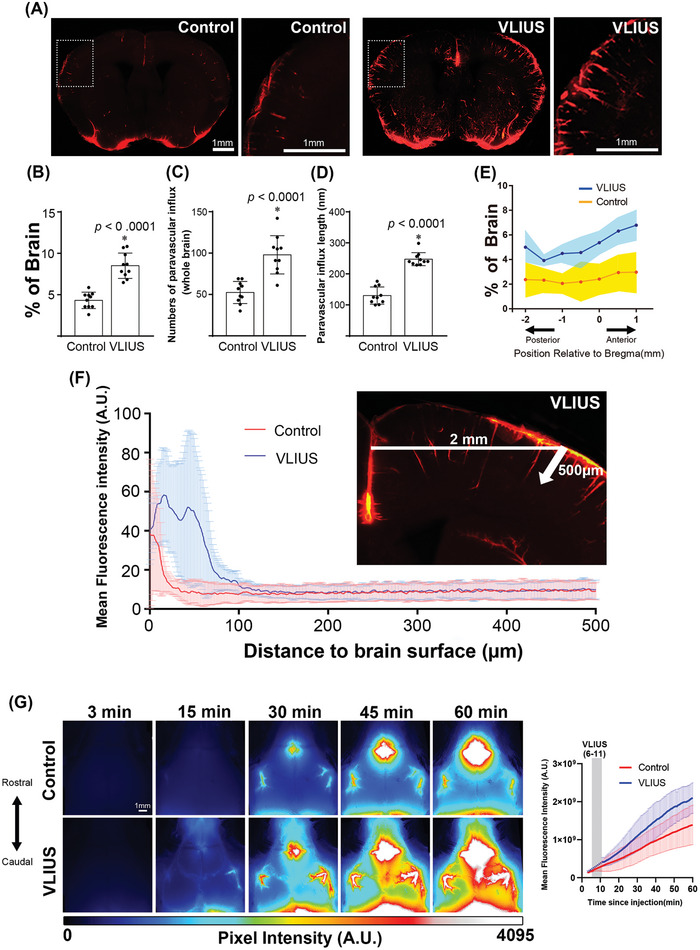
VLIUS stimulation increases CSF tracer influx. A) Representative images of coronal brain sections +0 mm from the bregma showing an increase in CSF tracer penetrance in response to very low‐intensity ultrasound (VLIUS) compared to controls. Scale bar: 1 mm. Quantification of the influx area B), influx numbers C), and influx length D) of VLIUS stimulation compared to controls: dots represent individual mice in each group. n = 10 mice/group; two independent repeats of n = 5 mice per group; E) Positional slice‐by‐slice representation of the area covered by tracer influx in coronal brain slices relative to the bregma. The solid line represents the percentage of the average tracer influx of all brain slices at that section per condition (shaded area = ±STDEV). n = 10 mice/group; two independent repeats of n = 5 mice per group F) Tracer penetration depth is measured at the cortical position 2 mm lateral to the midline, from the pial surface to a depth of 500 µm in the coronal section. Quantification of the mean fluorescence intensity of the tracer indicates that VLIUS stimulation induces greater penetration of the tracer deep into the brain (n = 5, respectively). G) Representative time‐lapse images of CSF influx over the first 60 min immediately following tracer injection in control and VLIUS‐stimulated mice. Images (16‐bit pixel depth) are color coded (royal form ImageJ) to depict pixel intensity (PI) in arbitrary units (AU). CSF, cerebrospinal fluid; VLIUS, very low intensity ultrasound. Scale bar: 1 mm. The results, for which the data are presented as mean ± SD (error bars denote SD), shown in Figure 1 (B, C, D) were analyzed using an independent t‐test to assess between‐group differences. An asterisk indicates *p* < 0.05.

First, we evaluated the effects of different ultrasound intensities on the circulation in the glymphatic system. The results showed that the intensity at the spatial peak temporal average intensity (I_spta_) of 3.68 mW cm^2^ could effectively promote tracer influx into the brain (**Figure** **1**A; Figure S2, Supporting Information). Furthermore, our utilization of 500 µm thick tissue, in conjunction with tissue‐clearing procedures, facilitated the acquisition of more intricate imaging results. These results allowed us to investigate specific aspects, including the extent and depth of cerebrospinal fluid (CSF) expansion within the paravascular spaces. We observed that VLIUS stimulation significantly increased both the quantity and depth of tracer diffusion into the paravascular spaces. (Figure 1B–D). Measurements of tracer penetrance at various slice positions relative to the bregma revealed that VLIUS stimulation increased CSF penetrance throughout the posterior (bregma −2) to the anterior (bregma +1) sections, indicating that the effect was not region‐specific (Figure 1E). Quantification of the tracer intensity in the cortical parenchyma indicated that depth‐dependent profiles decayed with cortical depth following the initial peak in both VLIUS‐stimulated and control mice. Notably, the tracer signals in the cortex of the VLIUS‐stimulated mice showed a higher intensity and deeper penetrance into the parenchyma (Figure 1F). In vivo transcranial live imaging of the CSF tracer revealed greater CSF influx in the VLIUS stimulation group compared with the control group at 15 min (Figure 1G and Video S1, Supporting Information). The tracer was distributed into the brain parenchyma through a network of paravascular spaces in the cerebral arteries on the brain surface. A greater degree of tracer infiltration into the brain was observed in VLIUS‐stimulated mice than in control mice at all observation times.

### 2.2 VLIUS Enhanced Interstitial Substance Clearance

**Figure 2:**
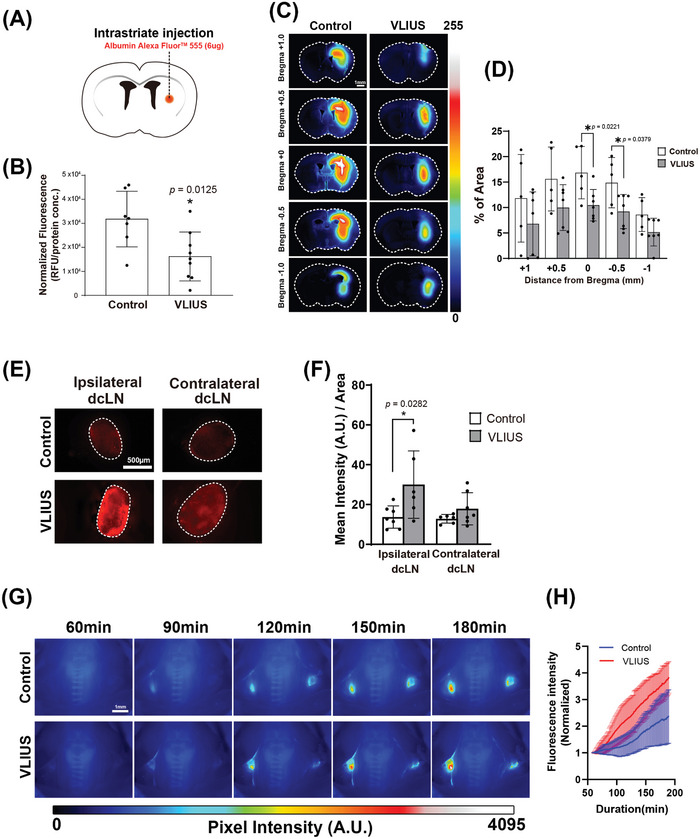
Increased interstitial fluid clearance observed through VLIUS stimulation. A) Schematic of the experiment. B) Three hours after injection, the remaining tracer is significantly lower in very low‐intensity ultrasound (VLIUS)‐stimulated mouse brains compared with controls (*p* = 0.0125, control group n = 7, VLIUS group n = 9), indicating that the clearance rate may be higher under VLIUS stimulation. Representative images C) and quantification of tracer area percentage D) in the coronal brain sections at different distances from the bregma reveal a reduction in the remaining tracer within the brain parenchyma in response to VLIUS compared to the control group. Scale bar: 1 mm. Representative images E) and quantification of tracer intensity F) of deep cervical lymph nodes (dcLN) three hours post‐intraparenchymal tracer injection showing a statistically significant increase in tracer intensity in the VLIUS group, indicating that the clearance rate may be higher under VLIUS stimulation. Scale bar: 500 µm. Representative time‐lapse images G) and quantification of tracer intensity H) from 60 to 180 min following tracer injection revealed a faster and higher increase in dcLN tracer intensity in the VLIUS group. Images (16‐bit pixel depth) are color coded (royal form ImageJ) to depict pixel intensity (PI) in arbitrary units (AU). Scale bar: 1 mm. The results, for which the data are presented as mean ± SD (error bars denote SD), shown in Figure 2 (B, D) and 2 (F) were analyzed using an independent t‐test and ANOVA followed by Tukey's post‐hoc test, to assess between‐group differences. An asterisk indicates *p* < 0.05.

In addition to promoting CSF tracer influx, we analyzed whether VLIUS promoted tracer removal from the anterior striatum. Three hours following the intrastriatal injection, the mice were euthanized, and the tracer residues in their brains were analyzed. VLIUS stimulation significantly reduced tracer residues in the injection area (**Figure** **2**B,C), indicating that VLIUS promotes waste clearance in the brain. The observed effect was not limited to a specific brain region, as evidenced by the significantly reduced tracer residues spanning the posterior (bregma −1) to the anterior (bregma +1) sections within the injection area (Figure 2C,D).

We further quantified the tracer intensity in the deep cervical lymph nodes (dcLNs), which revealed a marked elevation in the ipsilateral dcLNs (situated on the same side as the tracer injection site) following VLIUS stimulation (Figure 2E,F). In vivo transcranial live imaging revealed a faster and greater increase in dcLN tracer intensity in the VLIUS stimulation group compared with the control group (Figure 2G,H and Video S2, Supporting Information). Given that waste products within the brain may ultimately be cleared through the lymphatic vessels, ultimately reaching the dcLNs, this observation suggests that VLIUS stimulation facilitates the removal of substances from the brain.

### 2.3 VLIUS Promoted Glymphatic Function by Activating TRPV4

**Figure 3:**
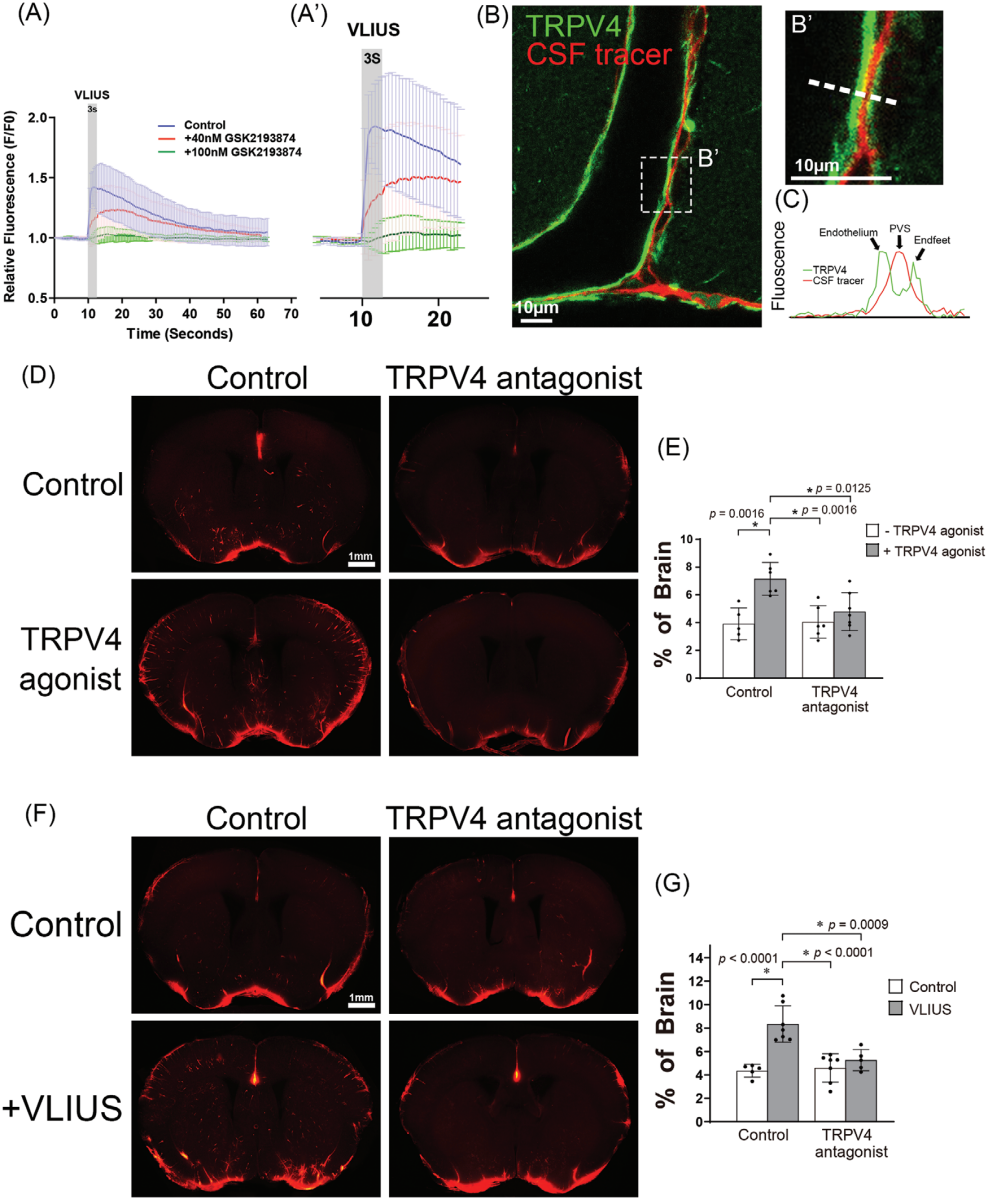
VLIUS promotes glymphatic function through the activation of TRPV4. A and A’) The calcium influx elevated by very low‐intensity ultrasound (VLIUS) stimulation was inhibited in a dose‐dependent manner by treatment with a transient receptor potential vanilloid‐4 (TRPV4) antagonist. B and B’) Along the cortical surface arteries, the cerebrospinal fluid (CSF) tracer (red) can be observed in the paravascular space, and TRPV4 (green) is expressed on endothelium and astrocytic endfeet. Scale bar: 10 µm. C) Representative images depicting fluorescence intensity projections from B'), indicated by white rectangles. D) The TRPV4 agonist (GSK1016790A) promoted CSF tracer permeability, which was inhibited by the co‐administered TRPV4 antagonist (GSK2193874). Scale bar: 1 mm. E) Quantification of the influx areas of various groups from D); the dots represent individual mice in each group (control group, n = 5; TRPV4 agonist group, n = 6; TRPV4 antagonist group, n = 6; and TRPV4 agonist + antagonist group, n = 7). F) VLIUS‐facilitated CSF permeability is inhibited by a TRPV4 antagonist. Scale bar: 1 mm. G) Quantification of the influx areas of various groups from F); the dots represent individual mice in each group (control group, n = 5; VLIUS group, n = 7; TRPV4 antagonist group, n = 7; and TRPV4 VLIUS + antagonist group, n = 5). Significant differences (analysis of variance with post hoc Tukey's test) are indicated with asterisks. The results, for which the data are presented as mean ± SD (error bars denote SD), shown in Figure 3(E) and (G) were analyzed using ANOVA followed by Tukey's post‐hoc test to assess between‐group differences. An asterisk indicates *p* < 0.05.

One prior study showed that mechanical waves (shock waves or ultrasound) could activate the TRPV4 mechanosensitive channel, thus promoting Ca^2+^ influx into vascular endothelial cells and ultimately affecting BBB integrity.^[^ ^17^ ^]^ We wished to determine whether VLIUS, at much lower intensities than those previously used, had similar effects on the TRPV4 channel. To achieve this, we used a micropipette‐guided ultrasound device^[^ ^21^ ^]^ to observe the effects of VLIUS stimulation on C6 cells in vitro. C6 cells are astrocyte‐like cells that endogenously express TRPV4. Astrocytes play an important role in the regulation of glymphatic function, while TRPV4 activation induces Ca^2+^ influx.^[^ ^22^ ^]^ Thus, the effects of VLIUS stimulation and TRPV4 antagonists on Ca^2+^ influx were investigated to determine whether VLIUS activated TRPV4. The results showed that VLIUS stimulation promoted Ca^2+^ influx, and that pretreatment with TRPV4 antagonists reduced these effects in a dose‐dependent manner (**Figure** **3**A).

In addition, TRPV4 is expressed in astrocytic endfeet to regulate Ca^2+^ oscillations, and to mediate vasodilation and vasoconstriction.^[^ ^23^ ^]^ The fact that cerebral artery pulsation drives the circulation of the glymphatic system,^[^ ^24^ ^]^ and that TRPV4 directly regulates vasodilation indicates that TRPV4 may affect glymphatic function. Our experiment revealed that the CSF tracer entered the paravascular space between the vascular endothelium and astrocyte endfeet, where TRPV4 was expressed (Figure 3B and B’), consistent with previous findings.^[^ ^23^, ^25^ ^]^ Treatment with a TRPV4 agonist (GSK1016790A) promoted tracer influx, and such effect was blocked by the co‐administration of a TRPV4 antagonist (GSK2193874). (Figure 3D,E) Similarly, VLIUS‐stimulated glymphatic influx was inhibited by the TRPV4 antagonist (GSK2193874) (Figure 3F,G), indicating the crucial role of TRPV4 in mediating VLIUS‐stimulated glymphatic influx.

### 2.4 AQP4 Water Channel Mediated TRPV4‐Facilitated Glymphatic Function

**Figure 4:**
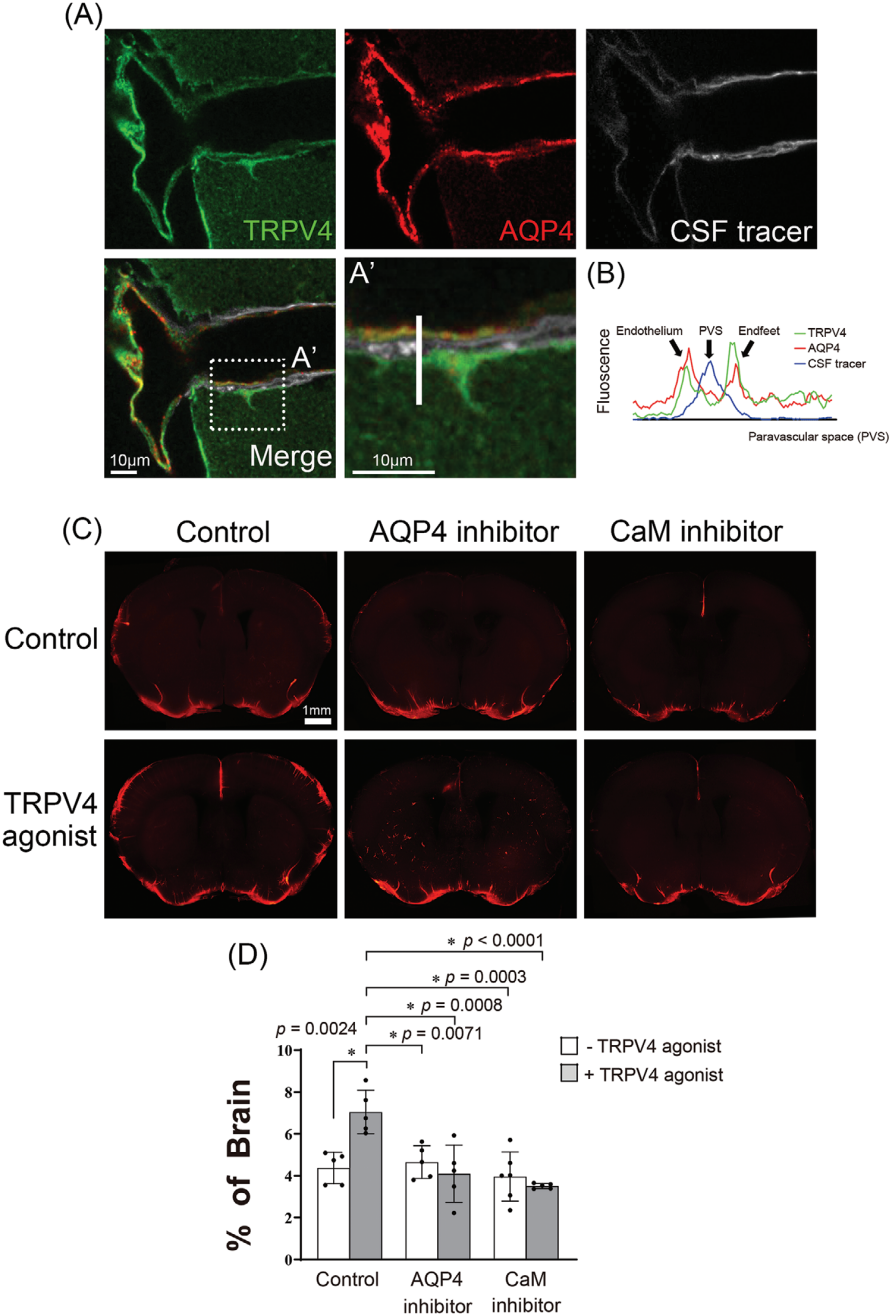
AQP4 is involved in TRPV4‐facilitated glymphatic circulation. A) Transient receptor potential vanilloid‐4 (TRPV4) and aquaporin‐4 (AQP4) co‐localized within the paravascular space of the adult mouse brain. In single‐plane confocal immunofluorescence images of cerebral surface artery, triple labeling with rabbit anti‐TRPV4 (green), mouse anti‐AQP4 (red), and cerebrospinal fluid (CSF) tracer (white) show the astrocyte endfeet and endothelial cells processes that are immunopositive for TRPV4 and AQP4. Scale bar: 10 µm. B) Representative images depict fluorescence intensity projections from A'), as indicated by a white line. Fluorescence imaging C) and quantification of the area covered by tracer influx D) in coronal brain slices show that 30 min after intracisternal injection, paravascular CSF influx increases with the administration of TRPV4 agonists, and does not increase with the co‐administration of the AQP4 inhibitor (AER271) or calmodulin inhibitor (trifluoperazine). The dots represent individual mice in each group (control group n = 5, TRPV4 agonist group n = 5, AQP4 inhibitor group n = 5, AQP4 inhibitor + TRPV4 agonist group n = 5, CaM inhibitor group n = 6, CaM inhibitor + TRPV4 agonist group n = 6). Scale bar: 1 mm. The results, for which the data are presented as mean ± SD (error bars denote SD), shown in Figure 4(D) were analyzed using ANOVA followed by Tukey's post‐hoc test to assess between‐group differences. An asterisk indicates *p* < 0.05.

AQP4 is an astrocytic water channel that plays an important role in regulating the glymphatic system, and contributes to the regulation of water homeostasis, waste clearance, neurotransmission, and response to brain injury.^[^ ^13^, ^26^ ^]^ TRPV4 and AQP4 synergistically regulate cellular and tissue functions such as astrocyte volume regulation,^[^ ^15^, ^27^ ^]^ calcium homeostasis,^[^ ^27^ ^]^ and CSF secretion,^[^ ^28^ ^]^ meaning that astrocytes are more sensitive to extracellular osmotic gradients.^[^ ^29^ ^]^ We hypothesized that AQP4 is involved in TRPV4‐promoted glymphatic circulation. The confocal analysis of mouse brain sections revealed that TRPV4 and AQP4 co‐localized at the astrocyte endfeet (**Figure** **4**A,A’,B). The TRPV4 agonist‐promoted CSF tracer influx was inhibited by concomitant administration of an AQP4 inhibitor (AER271) (Figure 4C,D).

Previous studies have shown that the TRPV4 channel facilitates the influx of Ca^2+^ into astrocytes, thus activating CaM, which then directly or indirectly increases the translocation of AQP4 to the cell surface to induce edema.^[^ ^14^, ^30^ ^]^ Trifluoperazine (TFP), a CaM antagonist, significantly inhibits AQP4 translocation to the cell surface in vitro and CNS edema.^[^ ^14^ ^]^ In this study, TFP treatment significantly decreased TRPV4 agonist‐induced CSF tracer influx (Figure 4C,D). Taken together, these findings indicate that TRPV4 agonist‐promoted glymphatic function is regulated by AQP4.

### 2.5 VLIUS‐promoted glymphatic function regulated by AQP4 water channels

**Figure 5:**
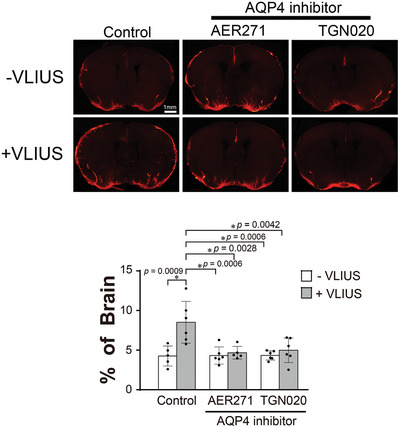
The role of AQP4 in VLIUS‐induced glymphatic circulation. Fluorescence imaging and quantification of the area covered by the tracer influx in coronal brain slices revealed that 30 min after intracisternal injection, paravascular cerebrospinal fluid influx increased with very low‐intensity ultrasound (VLIUS) stimulation, and did not increase with the co‐administration of an aquaporin‐4 (AQP4) inhibitor (AER271 and TGN020). Dots represent individual mice in each group (control group, n = 5; VLIUS group, n = 6; AQP4 inhibitor group, n = 6; and AQP4 inhibitor + VLIUS group, n = 5). Scale bar: 1 mm. The results, for which the data are presented as the mean ± SD (error bars denote SD), were analyzed using ANOVA followed by Tukey's post‐hoc test to assess between‐group differences. An asterisk indicates *p* < 0.05.

As previously described, VLIUS promotes glymphatic influx by activating TRPV4 (Figure 3D–G), while AQP4 is required to regulate the influx of CSF tracers into the brain parenchyma following TRPV4 activation (Figure 4C,D). We subsequently investigated whether VLIUS‐promoted CSF tracer influx was similarly mediated by the AQP4 water channel. Concomitant administration of an AQP4 inhibitor (either AER271 or TGN020) significantly inhibited the VLIUS‐induced CSF tracer influx (**Figure** **5**). These results indicate that the VLIUS‐TRPV4‐AQP4 pathway plays a significant role in modulating the glymphatic system.

### 2.6 VLIUS Facilitates the Clearance of Exogenous β‐Amyloid, Mediated by TRPV4

**Figure 6:**
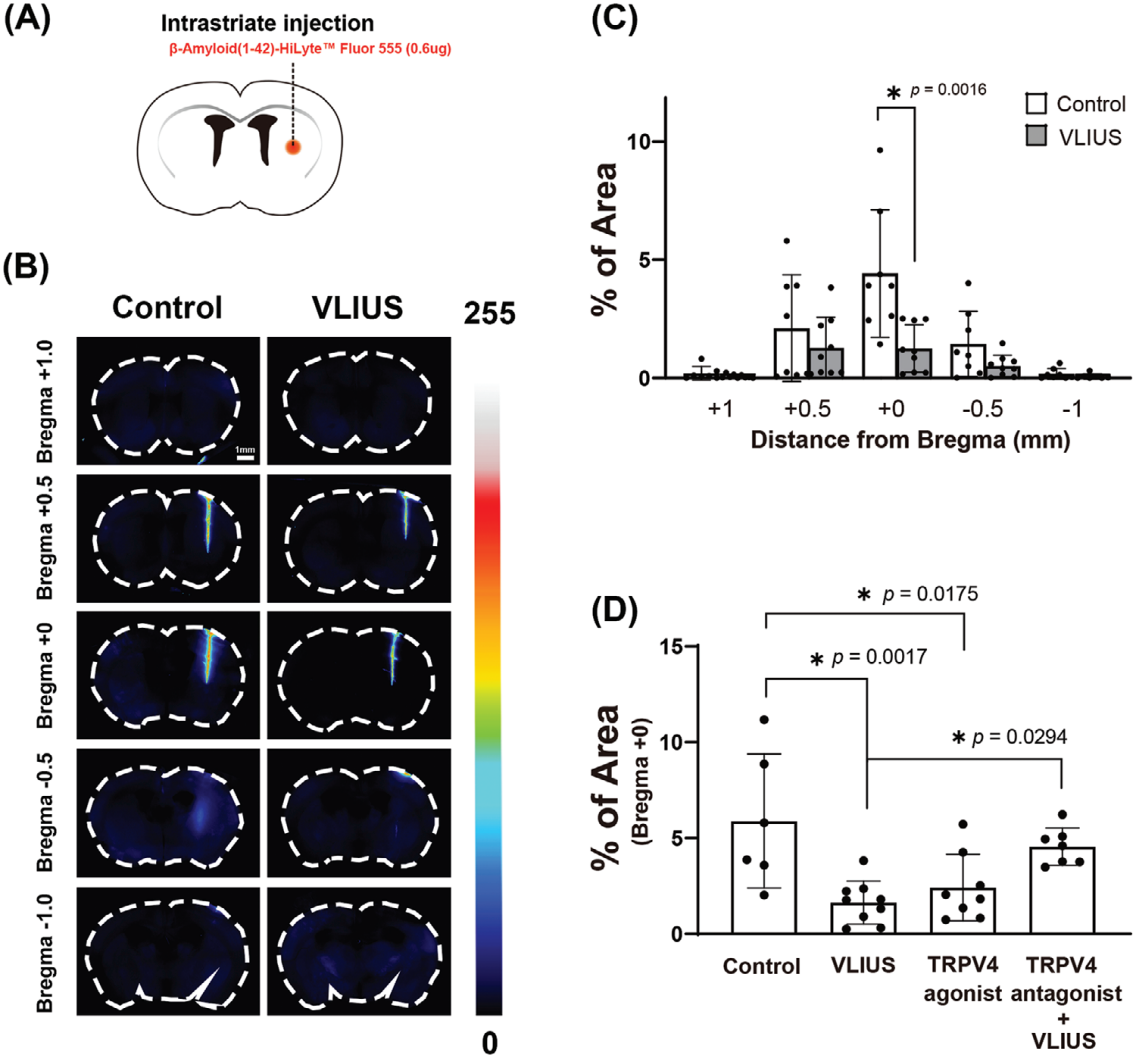
TRPV4 is involved in the VLIUS‐facilitated clearance of β‐amyloid (1‐42). A) Schematic of the experiments. Representative images B) and quantification of β‐amyloid (1‐42) area percentage C) in coronal brain sections at different distances from the bregma reveal a reduction in the β‐amyloid (1‐42) remaining within the brain parenchyma in response to VLIUS compared to the control group. Scale bar: 1 mm. D) Both VLIUS and the TRPV4 agonists (GSK1016790A) promoted β‐amyloid (1‐42) clearance. VLIUS‐promoted clearance was inhibited by co‐administration of a TRPV4 antagonist (GSK2193874). Dots represent individual mice in each group (control group, n = 6; VLIUS group, n = 9; TRPV4 agonist group, n = 8; and TRPV4 antagonist + VLIUS group, n = 7). The results, for which the data are presented as the mean ± SD (error bars denote SD), shown in Figure 6(C) and (D) were analyzed using the Mann‐Whitney U test and ANOVA followed by Tukey's post‐hoc test, respectively, to assess between‐group differences. An asterisk indicates *p* < 0.05.

In healthy brains, β‐amyloid is naturally expressed and cleared through the glymphatic system. However, impairments in the clearance of β‐amyloid can lead to its accumulation and, ultimately, plaque aggregation, which can disrupt neuronal function and cause cell death. In this study, we demonstrated that VLIUS enhanced the clearance of fluorescent tracers; however, whether it could aid in the removal of β‐amyloid is crucial to understand the future clinical therapeutic application of VLIUS. Following intrastriatal injection of β‐amyloid (1‐42), mice were euthanized, and the β‐amyloid residues in their brains were analyzed. VLIUS stimulation significantly reduced β‐amyloid residues in the injection area (**Figure** **6**B,C), indicating that VLIUS improves β‐amyloid clearance in the brain. Furthermore, treatment with a TRPV4 agonist promoted β‐amyloid clearance, while treatment with a TRPV4 antagonist attenuated VLIUS‐mediated β‐amyloid clearance (Figure 6D). These results strongly suggest that VLIUS plays a role in facilitating the removal of waste products from the brain, in a manner mediated by TRPV4.

### 2.7 VLIUS Stimulation Promotes AQP4 Translocation to the Cell Surface

Previous studies have shown that TRPV4 activation promotes Ca^2+^ influx, subsequently facilitating the translocation of AQP4 to the cell surface.^[^ ^14^, ^30^ ^]^ Building on this, our results provide evidence that VLIUS activates TRPV4, resulting in an influx of Ca^2+^ into the cells (Figure 3A). Three different experiments were conducted to investigate whether VLIUS enhanced the translocation of AQP4 to the cell surface through the TRPV4‐mediated pathway.

**Figure 7:**
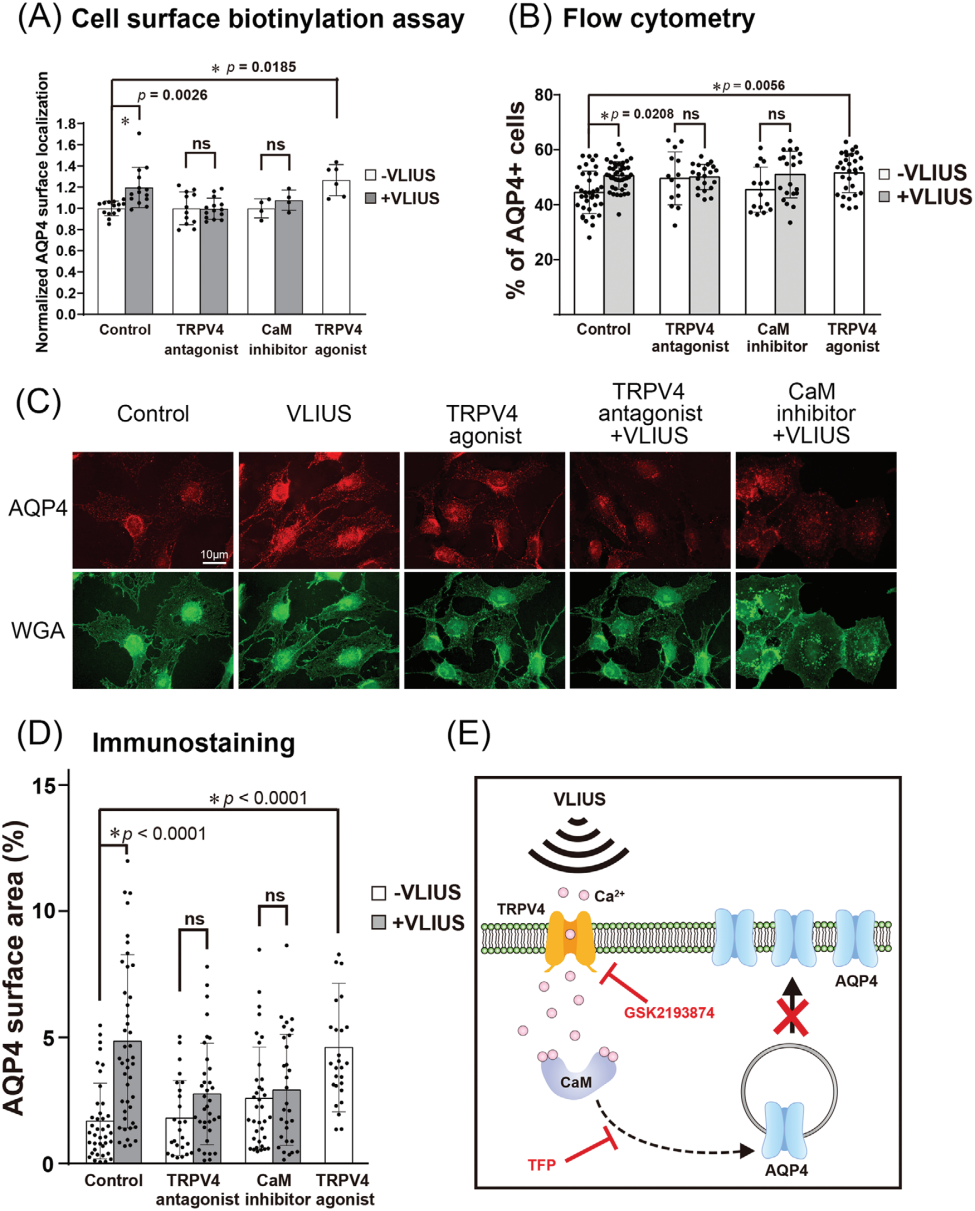
AQP4 protein translocation to the cell surface increased after VLIUS stimulation. A) The mean fold change in aquaporin‐4 (AQP4) surface expression, measured by cell‐surface biotinylation in C6 cells. The calmodulin (CaM) inhibitor was 20.8 µM trifluoperazine (TFP). The transient receptor potential vanilloid‐4 (TRPV4) antagonist was 100 nM GSK2193874, while the TRPV4 agonist was 2 µM GSK1016790A. Cells had been pre‐incubated with drug 30 min before very low‐intensity ultrasound (VLIUS) treatment. AQP4 on the cell surface significantly increases after treatment with VLIUS or TRPV4 agonist. However, administration of TRPV4 antagonist or CaM inhibitor inhibits VLIUS‐facilitated AQP4 cell surface localization. B) The AQP4 surface expression is analyzed by flow cytometry (without permeabilization). The population of AQP4‐positive cells was significantly increased by VLIUS or TRPV4 agonists. Administration of TRPV4 antagonist or CaM inhibitor inhibits the VLIUS effect. C) AQP4 on the cell surface can also be observed by immunofluorescence staining (without permeabilization). Cells had been counterstained by WGA‐conjugated Alexa Fluor™ 488 to determine cell boundaries. Scale bar: 10 µm. D) Quantification of AQP4 on the cell surface using immunofluorescence staining. E) The schematic showing that both the CaM inhibitor and the TRPV4 antagonist can inhibit VLIUS‐induced AQP4 translocation to the cell surface. The results, for which the data are presented as the mean ± SD (error bars denote SD), shown in Figure 7 (A,B,D) were analyzed using Kruskal‐Wallis test followed by Dunn's post‐hoc test to assess between‐group differences. An asterisk indicates *p* < 0.05.

First, the cell‐surface expression of AQP4 after 30 min of VLIUS treatment was compared with that in control cells using a cell surface biotinylation assay. The results showed that AQP4 levels on the cell surface were significantly increased by both VLIUS treatment and treatment with the TRPV4 agonist (GSK1016790A) (**Figure** **7**A). Because Ca^2+^ influx‐activated CaM is a key regulator of AQP4 translocation,^[^ ^14^, ^30^ ^]^ we measured the localization of AQP4 following the inhibition of either CaM (using TFP) or TRPV4 (using GSK2193874). The results showed that treatment with the CaM inhibitors and TRPV4 inhibitors decreased surface AQP4 levels to control levels, thus reducing the effect of VLIUS on AQP4 translocation (Figure 7A).

Next, we used flow cytometry to analyze the proportion of AQP4^+^ cells on the surface. The cells were only fixed with 4% paraformaldehyde (PFA) without TritonX‐100 treatment prior to immunostaining to ensure that only the AQP4 proteins on the cell surface were detected. Both VLIUS stimulation and TRPV4 agonists increased the population of AQP4^+^ cells. Conversely, TRPV4 antagonists and CaM inhibitors attenuated the VLIUS‐induced translocation of AQP4 (Figure 7B).

Third, we observed the expression of AQP4 on the cell surface using fluorescence microscopy in the absence of TritonX‐100 treatment, further performing wheat germ agglutinin (WGA) staining to determine the boundary of the cells. The intensity and proportion of AQP4 on the cell surface significantly increased following treatment with VLIUS or the TRPV4 agonist (Figure 7C,D).

The combined results of the above three experiments indicated that VLIUS increased AQP4 protein translocation to the cell surface, in a manner potentially mediated by TRPV4 and calmodulin.

### 2.8 VLIUS Stimulation Modulated Astrocytic Cell Volume within Glia Limitans

Overall, we found that VLIUS stimulation promoted AQP4 translocation to the cell surface. We therefore sought to understand the implications of this phenomenon on cellular behavior. AQP4 mediates water influx to induce cell swelling, and triggers a regulatory volume decrease (RVD) to restore the original volume in response to swelling, suggesting that AQP4 is involved in regulating cell volume.^[^ ^31^ ^]^ Moreover, the presence of abundant AQP4 on the cell surface has been associated with cytotoxic edema, while the inhibition of AQP4 has been shown to ameliorate this edema, resulting in improved electrophysiological, sensory, and locomotor functions in animal models.^[^ ^14^ ^]^ Since AQP4 was translocated to the cell surface after VLIUS stimulation, we investigated whether VLIUS induced cell volume changes and led to persistent cytotoxic edema.

**Figure 8:**
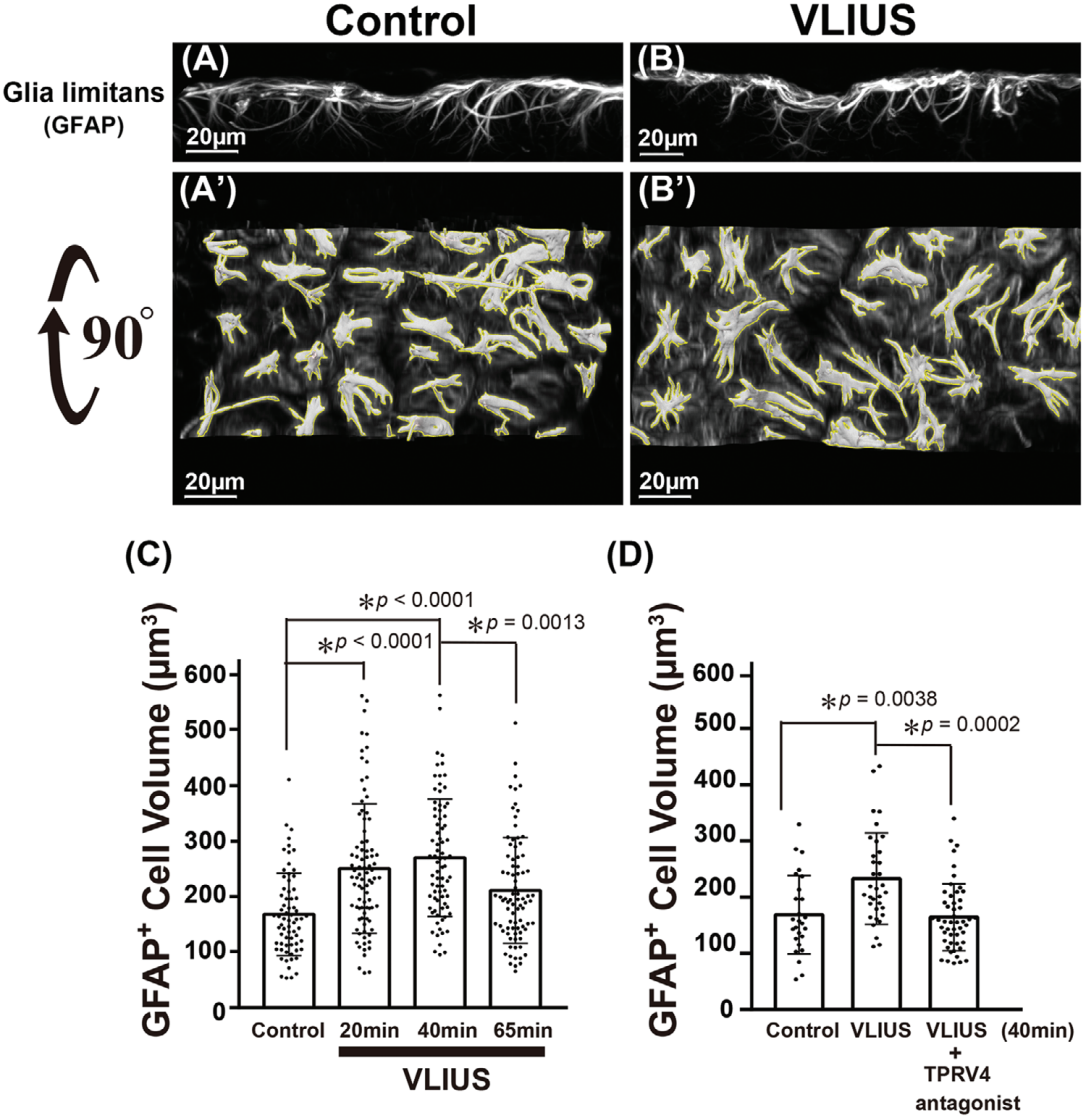
The volume of astrocytes in the glia limitans are altered following VLIUS stimulation. Reconstruction of 3D images from confocal microscopy using Imaris software, and are shown as control A), very low‐intensity ultrasound (VLIUS) B), and 90° rotated images A') and B'). Scale bar: 20 µm. The yellow line represents the analysis of each intact cell (with the same threshold) using the surface creation wizard of Imaris software. Quantification shows that cell volume was altered by VLIUS stimulation C), and this effect was blocked by a transient receptor potential vanilloid‐4 (TRPV4) antagonist D). Dots represent individual intact cells in each group. The results, for which the data are presented as the mean ± SD (error bars denote SD), shown in Figure 8 (C) and (D) were analyzed using the Kruskal‐Wallis test followed by Dunn's post‐hoc test, and ANOVA followed by Tukey's post‐hoc test, respectively, to assess between‐group differences. An asterisk indicates *p* < 0.05.

In this experiment, we observed glial fibrillary acidic protein (GFAP)‐positive astrocytes in the glia limitans (**Figure** **8**A,B). We selected the glia limitans as our focus, as opposed to the paravascular astrocyte endfeet, for several reasons: 1) the spatial structure of the endfeet presents challenges in terms of observation, distinction, and analysis; 2) the astrocytic endfeet surrounding the blood vessels are extensions of the glia limitans; and 3) we observed that diffusion of the CSF tracer occurred from the brain surface to the brain parenchyma, in addition to from the paravascular space (Figure S3, Supporting Information). Furthermore, it has been reported that the glia limitans contains astrocytes with high GFAP expression.^[^ ^32^ ^]^

We attempted to understand the role of AQP4 translocation to the cell surface by observing the changes in the volume of GFAP‐positive astrocytes. The volume of GFAP‐positive cells significantly increased from 20 and 40 min after VLIUS stimulation, subsequently decreasing significantly 65 min after VLIUS stimulation (Figure 8A–C). In other words, the volume of GFAP‐positive cells increased transiently (≈20–40 min) following VLIUS stimulation and was later restored, indicating that VLIUS stimulation did not induce persistent cytotoxic edema. Furthermore, we did not observe any harmful effects of VLIUS stimulation (Figure S4, Supporting Information).

Notably, both TRPV4 in isolation^[^ ^33^ ^]^ and in combination with AQP4 ^[^ ^15^, ^27^ ^]^ have been implicated in the modulation of cell volume and the RVD mechanism. Additionally, the co‐localization of TRPV4 expression with GFAP has been observed in the glia limitans.^[^ ^34^ ^]^ In the current study, we found that TRPV4 activation enhanced AQP4 translocation to the cell surface (Figure 7), and that the TRPV4 antagonist significantly inhibited the VLIUS‐induced increases in cell volume (Figure 8D). These findings collectively indicate that VLIUS‐induced TRPV4 activation may facilitate the translocation of AQP4 to the cell surface, thus modulating the movement of water in and out of the cell and consequently altering cell volume.

### 2.9 No Observable Side Effects of VLIUS Stimulation

In safety experiments, the VLIUS showed no observable brain damage. As shown in Figure S4 (Supporting Information), H&E staining did not reveal tissue damage, gliosis, or chromatolysis. Furthermore, Luxol Fast Blue staining combined with Nissl staining did not show any demyelination, loss of Nissl bodies (indicating neuronal abnormalities), or chromatolysis. NeuN and GFAP immunostaining showed no significant decrease in the number of neurons or any abnormal increase in the number of astrocytes.

Furthermore, we tested the effect of VLIUS on the BBB, using the Evans blue extravasation experiment to illustrate this. In this experiment, the mice were injected with Evans blue via the tail vein immediately after VLIUS stimulation and sacrificed 30 min after VLIUS stimulation. The results showed that VLIUS stimulation did not cause Evans blue leakage from the blood vessels into the brain tissue (Figure S5, Supporting Information). Furthermore, immunostaining for tight junction‐related proteins showed that the co‐localization of ZO1‐Claudin5 and ZO1‐Occludin was not affected (Figure S5C, Supporting Information). Taken together, these results indicated that VLIUS stimulation did not cause any observable damage to the BBB.

## 3 Discussion

The primary observation of this study was the enhancement of glymphatic influx induced by VLIUS, potentially through the involvement of the TRPV4‐CaM‐AQP4 pathway within astrocytes. TRPV4 is activated by VLIUS in astrocytes, inducing an influx of Ca^2+^ that activates CaM. This in turn promotes the translocation of AQP4 to the cell surface, leading to water influx and increased cell volume. Sixty‐five minutes after VLIUS stimulation, the swollen cells were restored to their original volume, potentially due to water efflux. This transcellular water flow may be the driving force behind VLIUS‐stimulated glymphatic circulation.

The glymphatic system is considered an important target for therapeutic intervention in CNS disorders.^[^ ^19^, ^35^ ^]^ Ultrasound, owing to its noninvasiveness and availability, has been applied for the treatment of various CNS disorders, with outcome effects primarily focusing on opening the BBB. However, the incident ultrasound intensity or pressure (pressure level of ≈300–800 kPa^[^ ^36^ ^]^) is usually much higher than that used in the present study. Furthermore, most studies have included the addition of microbubbles to generate cavitation and open the BBB. Therefore, the risk of brain injury is increased. Irreversible damage may occur if the generated cavitation is too intense, or if a patient's blood vessels or BBB structures are too fragile.^[^ ^37^ ^]^ One recent study demonstrated that focused ultrasound combined with microbubbles enhances glymphatic transport within the brain.^[^ ^38^ ^]^ In the present study, a planar ultrasound transducer was used with a relatively low intensity (I_spta_:3.68 mW cm^2^, pressure level 98 kPa). As previously shown, even low energy can generate various bioeffects, such as promoting adult neurogenesis in the dentate gyrus.^[^ ^20^ ^]^ Another study focusing on neurogenesis in the dentate gyrus revealed that the group without microbubbles had better bioeffects and functional recovery than the group treated with microbubbles,^[^ ^39^ ^]^ indicating that microbubbles are not necessary for brain stimulation. Another study showed that focused ultrasound (at a pressure level that did not cause BBB disruption) without the use of microbubbles promoted the transport of intracortically injected tracers, and facilitated their clearance from the brain.^[^ ^40^ ^]^ When converted to our measurements, the peak‐to‐peak pressure of 770 kPa in this prior study^[^ ^40^ ^]^ corresponded to 385 kPa, whereas our study used ≈98 kPa, which was significantly lower. The current study revealed that VLIUS without microbubbles enhanced glymphatic influx while exerting no harmful effects on the brain were observed (Figures S4 and S5, Supporting Information). VLIUS without microbubbles is a safe and effective method to modulate the glymphatic system.

The TRPV4‐CaM‐AQP4 pathway may be one of the mechanisms by which VLIUS enhances glymphatic function. It is important to note that there is a degree of complexity surrounding the role of TRPV4 in conjunction with AQP4. One study indicated that TRPV4 interacts with AQP4 to regulate the entry and exit of water molecules into and out of cells to regulate cell volume, which promotes the influx of Ca^2+^ by driving TRPV4 activation and subsequently regulates RVD in hypotonic environments.^[^ ^15^ ^]^ Conversely, another study proposed that TRPV4‐induced Ca^2+^ influx may not be the primary factor in RVD regulation, as TRPV4 blockade or the removal of external Ca^2+^ did not prevent RVD in hypotonic environments.^[^ ^31b^ ^]^ In this study, AQP4‐M23 was overexpressed to observe changes in cell volume, allowing water to enter the cells spontaneously.^[^ ^31b^ ^]^ It is important to consider that AQP4 has multiple isoforms that are present in varying proportions within cells and that play distinct roles,^[^ ^31a^ ^]^ suggesting that the overexpression of AQP4‐M23 alone may not provide a complete explanation. Additionally, the response generated by the residual Ca^2+^ cannot be entirely discounted.^[^ ^31b^ ^]^ For example, previous studies have suggested that TRPV4 induces Ca^2+^ oscillations in astrocytic endfeet to regulate neurovascular coupling.^[^ ^23^ ^]^ Activated TRPV4 promotes external Ca^2+^ influx into cells and triggers the activation of inositol trisphosphate receptors, amplifying local Ca^2+^ signals, and initiating Ca^2+^ waves.^[^ ^15^ ^]^ A small Ca^2+^ influx is sufficient to amplify Ca^2+^ signals and form subsequent Ca^2+^ oscillations. As such, it is generally considered that the synergistic effect of TRPV4 and AQP4 regulates cell volume and increases the sensitivity of astrocytes to environmental stress.^[^ ^14^, ^29^ ^]^ However, these previous studies were based on hypotonic stress, which activates TRPV4 owing to changes in cell membrane tension caused by AQP4, thereby facilitating water entry into cells. The ultrasound‐generated mechanical force used to activate TRPV4 may have differed from the hypotonicity‐induced changes in membrane tension.

The activation of TRPV4 by agonists or VLIUS can promote the transport of AQP4 from the cytosol to the cell surface (Figure 7)^[^ ^14^, ^30^ ^]^ which can effectively promote CSF tracer influx, whereas TRPV4 blockade significantly inhibits VLIUS‐promoted CSF tracer influx (Figure 3). Our results also showed that the effects of VLIUS stimulation were better than those of TRPV4 agonists alone, suggesting that, in addition to TRPV4 receptors, other mechanosensitive channels sensitive to VLIUS stimulation may be present in astrocytes.^[^ ^41^ ^]^ Moreover, prolonged activation of TRPV4 by agonists can lead to irreversible side effects, such as a reduced expression of tight junction proteins,^[^ ^42^ ^]^ BBB disruption,^[^ ^42b^ ^]^ and apoptosis induction.^[^ ^43^ ^]^ Unlike CNS injury, VLIUS stimulation does not cause irreversible cytotoxic edema. The increased cell volume stimulated by VLIUS returned to the baseline 65 min after stimulation (Figure 8). The tissue staining assays did not reveal any tissue disruption (Figure S4, Supporting Information). Therefore, VLIUS may be preferable for promoting glymphatic circulation.

Previous studies have indicated that glymphatic function is accelerated by neurovascular coupling.^[^ ^44^ ^]^ Interestingly, TRPV4, which is expressed in vascular endothelial cells, vascular smooth muscle cells, and astrocyte endfeet, can amplify neurovascular coupling responses when activated,^[^ ^23a^ ^]^ and is considered a critical regulator of vasodilation. Vasodilation affects intracranial arterial pulsation, which is believed to be the primary driving force of the glymphatic system.^[^ ^24^ ^]^ In our in vivo real‐time imaging study, we observed that the VLIUS directly accelerated cerebral blood flow (unpublished data), indicating that it may mediate neurovascular coupling. As such, it is reasonable to speculate that the VLIUS activates TRPV4, which amplifies neurovascular coupling, thus improving the glymphatic system. Given that TRPV4 mediates vasodilation and may interfere with arterial pulsation, it is plausible that the mechanical forces driving the glymphatic influx are modulated by TRPV4 activity. This suggests that ultrasound may enhance glymphatic function, not only by increasing AQP4‐mediated influx, but also by modulating vascular dynamics. However, this hypothesis requires further investigation.

Overall, our findings have several significant clinical implications. Given the limited activity of the glymphatic system during awakening, the incorporation of the VLIUS into a wearable device could offer continuous enhancement of glymphatic clearance, even upon awakening. Importantly, our results demonstrated that VLIUS, when applied without microbubbles, effectively enhanced glymphatic function in mice without inducing brain injury. The integration of the VLIUS into wearable devices further represents a potential therapeutic avenue for CNS disorders associated with glymphatic dysfunction. However, a potential challenge for this application lies in accounting for variations in skull thickness and brain volume in humans. While we observed only a 20% decrease in VLIUS energy after traversing the mouse skull (Figure S6, Supporting Information), adjusting specific VLIUS parameters is imperative to ensure positive effects on the human CNS. Augmenting the intensity and treatment duration may be necessary, although the potential risks associated with such modifications require a thorough examination.

This study had several limitations. First, the lack of availability of AQP4‐specific agonists precluded our ability to confirm whether the activation of AQP4 affects TRPV4, or vice versa. However, the direction of the relationship between these two key molecular players remains unclear. Second, the use of postmortem samples poses inherent challenges, as the associated collapse of arteries and the subsequent disappearance of fluid in the paravascular space could lead to potential misjudgments in observations.^[^ ^26^ ^]^ Employing transgenic mouse models with fluorescently labeled astrocytes coupled with two‐photon microscopy could facilitate more detailed visualization allowing a better understanding of the spatiotemporal dynamics within the glymphatic system.^[^ ^45^ ^]^ Third, although the current study demonstrated that VLIUS stimulation is associated with TRPV4‐mediated calcium influx, the direct link between ultrasound stimulation and TRPV4 activation remains to be elucidated. Further investigations are therefore required needed to delineate the specific mechanotransductive signaling pathways involved in this process and how they contribute to the observed enhancement of glymphatic function.

Nevertheless, our results did not fully explain the exact mechanism underlying RVD modulation. It is speculated that the driving force generated by Cl^−^ efflux via the activation of downstream volume‐regulated anion channels or calcium‐activated chloride channels causes water efflux and reduces cell volume.^[^ ^28^, ^46^ ^]^ TRPV4, AQP4, and anoctamin 1 all cooperate to regulate water efflux and the cell volume of choroid plexus epithelial cells (CPECs), suggesting their role in CSF production.^[^ ^28a^ ^]^ Furthermore, TRPV4 appears to be a cell surface sensor that promotes cell secretion after receiving extracellular stimuli. The activation of TRPV4 promotes the exocytosis of various molecules, including AQP4 (in astrocytes),^[^ ^14^ ^]^ TRPV4 itself (in human umbilical vein endothelial cells),^[^ ^47^ ^]^ and alpha‐klotho, sodium, and potassium‐adenosine triphosphatase (in CPECs).^[^ ^48^ ^]^ Upon stimulation, TRPV4 is activated to promote the secretion of water or proteins from CPECs into the extracellular space, thus indicating that TRPV4 may regulate CSF secretion. Since TRPV4 is abundantly expressed in the choroid plexus, VLIUS stimulation may promote CSF secretion. Further studies are warranted to investigate these points.

## 4 Experimental Section

### Animals

All animal experiments were performed in accordance with the guidelines of the Institutional Animal Care and Use Committee of the National Taiwan University College of Medicine (approval no. 20 201 028). C57BL/6JNarl mice (body weight, 20—25 g) were purchased from the National Laboratory Animal Center (Taiwan), and maintained under a controlled 12 h dark light/12 h cycle with access to water and food ad libitum. In all experiments, animals were anesthetized using a combination of Zoletil (50 mg kg^−1^, intraperitoneally) and xylazine (2.3 mg kg^−1^, intraperitoneally).

### Ultrasound Devices and Stimulation Parameters

The ultrasound device setup was described in our previous study.^[^ ^20^ ^]^ The ultrasound field was generated using a 1 MHz commercial plane transducer (C539‐SM; Olympus, Tokyo, Japan) for mouse brain stimulation. All stimulations were performed using a function generator (Tektronix AFG1022, Beaverton, OR, USA), equipped with a power amplifier (E&I 210 L, Electronics & Innovation, Rochester, USA). The planar transducer was placed at the center of the brain, and an ultrasound gel was applied at the interface between the bottom of the transducer and the mouse scalp. Following intracisternal injection, the mice were immediately stimulated with VLIUS for 5 min. VLIUS treatment was performed under the following conditions: center frequency, 1 MHz; pulse repetition frequency (PRF), 1 kHz; duty factor, 1%; and various spatial peak temporal average intensities (I_spta_) of 0.92, 3.68, and 5.85 mW cm^2^ (Figures S1 and S2, Supporting Information). Among these, I_spta_ = 3.68 mW cm^2^ (pressure level of ≈98 kPa) was the optimal condition for promoting tracer diffusion (Figure S2, Supporting Information); as such, the subsequent experiments were conducted under these conditions. A more specific parameter was the pulse repetition period (1/PRF), which differs from the typical setting of BBB opening. The period was shortened such that it appeared similar to a continuous wave when viewed macroscopically. However, because the duty cycle was only 1%, it prevents heat accumulation and the generation of cavitation. Compared to the commonly used ultrasound parameters, which remain at 10^4^ cycles/s, the VLIUS parameter was distributed within 1 s as 10 cycles/pulse completed in 1000 pulses,^[^ ^20^ ^]^ whereas the typical ultrasound parameter setting completes it in 10^4^ cycles/pulse with one pulse.

For in vitro experiments, 1 × 10^5^ cells were seeded in a 24‐well plate 18 h before the experiments. After renewing the medium, a planar transducer was placed directly above the cells. The VLIUS setting was the same as that described above, except that the duration was changed to 1 min. One hour following VLIUS stimulation, the cells were washed with phosphate buffered saline (PBS) in preparation for subsequent experimentation. For information regarding micropipette‐guided ultrasound stimulation, please refer to the section on live‐cell calcium signal imaging.

### Intracisternal CSF Tracer Infusion

Intracisternal injection was performed as previously described with minor modifications.^[^ ^49^ ^]^ The mice were weighed and anesthetized by an intraperitoneal injection of Zoletil/xylazine. The cisterna magna was exposed through a surgical incision using a stereotactic frame, while a 30‐gauge needle connected to a Hamilton syringe via a polyethylene tube (PE10) was inserted into the cisterna magna. Fluorescent CSF tracers (Alexa Fluor 555; Cat No. A34786; Thermo Fisher Scientific, Waltham, MA, USA) were added to the artificial CSF at a concentration of 5 mg ml^−1^. These CSF tracers (6 µl) were infused at a constant rate of 1 µl min^−1^ with a syringe pump (Harvard Apparatus, Holliston, MA, USA). During the experiment, the needle was kept in place to avoid depressurization of the CSF compartment. In the VLIUS stimulation group, mice were immediately stimulated with VLIUS for 5 min after intracisternal infusion. Thirty minutes after the start of intracisternal infusion, the mice were euthanized, and brains were harvested and fixed with 4% cold paraformaldehyde in PBS overnight. To evaluate the distribution of tracers into the brain parenchyma, 500 µm coronal slices were cut on a vibratome (MicroSlicer™ DTK‐1000N, DSK, Kyoto, Japan) and then incubated in 2% phosphate‐buffered saline with Tween (PBST) (2% Triton X‐100 in PBS) for 2 days to promote tissue permeability. After washing three times with PBS, the tissues were cleared and incubated with RapiClear® 1.49 (SunJin Lab Co., Hsinchu City, Taiwan) for 1 h to promote transparency and were then mounted with fresh RapiClear®. Samples were imaged using a fluorescence microscope (Olympus BX51 with a ToupTek camera) and the extended depth‐of‐field (EDF) method (ToupView software, Hangzhou, China) to provide higher‐quality images and ensure faster acquisition of large amounts of information. The tracer influx (area %) was analyzed using ImageJ software (16‐bit image type; analyze particle setting: 80–infinity µm^2^ size range, 0.0–1.0 circularity, and automatic threshold). The number of paravascular influxes was counted in brain sections where the subarachnoid tracer extended into the parenchyma (Figure 1C). The influx length was further measured from the subarachnoid space to the distal end using the ToupView software (Figure 1D). Additionally, 100 µm brain slices instead to measure the diffusion of the tracer in the anterior to posterior regions of the brain was used, for more refined results (Figure 1E).

### Transcranial Live Imaging

For in vivo transcranial live imaging, the skin covering the dorsal calvarium was incised and a tracer was injected through the cisterna magna. The entry of the CSF tracers into the brain was imaged using fluorescence macroscopy (Olympus MVX10, Tokyo, Japan) with an ORCA‐Spark digital CMOS camera (C11440‐36U, Hamamatsu, Japan). Images were recorded at 30 s intervals for 0—64 min following injection commencement using the CellSens Standard 3 (Olympus). The exposure time was the same throughout the imaging sequence and across all experimental groups.

### Intraparenchymal Injection of Tracer and β‐amyloid

To evaluate the clearance rates of interstitial fluid from the brain after VLIUS treatment, the tracer (5 mg ml^−1^) or human β‐amyloid (1‐42)‐HiLyte™ Fluor 555‐labeled (0.5 mg ml^−1^) (Cat No. AS‐60480; ANASPEC, Fremont, CT, USA) were stereotactically injected into the brain parenchyma. The anesthetized mice were fixed in a stereotaxic frame, and a 30G needle was inserted via a burr hole into the left striatum (0.22 mm caudal, 2.5 mm lateral, 3.5 mm ventral to bregma). The tracer or human β‐amyloid (1‐42) (total 1.2 µl) was injected at a rate of 200 nl min^−1^, following which the needle was maintained in place for an additional 30 min. After intrastriatal injection, the mice were treated with or without VLIUS stimulation, after which a 3 h waiting time for the tracer or β‐amyloid to be cleared from the parenchyma through the interstitial solute clearance mechanism. After 3 h, the mice were euthanized, and their brains were harvested. For quantitative analysis, brain tissues were homogenized on ice with the Pierce^TM^ RIPA Buffer (Cat No. 89 900, Thermo Fisher Scientific), then centrifuged at 12 000 g at 4 °C for 20 min. The supernatants were then collected and measured using a microplate reader (Infinite M200; Tecan, Austria). The tracer content is shown as the relative light units per milligram of protein. The protein concentration was measured using the Pierce™ Coomassie Protein Assay (Cat No. 1 856 209, Thermo Fisher). For image analysis, the brain was sectioned into 500 µm coronal slices as described above, and images were recorded after treatment with Triton and RapiClear®. The tracer or β‐amyloid distribution within the brain was subsequently analyzed using ImageJ software to determine its proportion.

### Deep Cervical Lymph Node Collection for Waste Clearance Analysis

Three hours post‐intraparenchymal tracer injection, the deep cervical lymph nodes (dcLNs) were harvested and subsequently fixed overnight in 4% cold paraformaldehyde in PBS. Subsequently, the dcLNs were immersed in RapiClear®, and images were recorded as described previously. The tracer content within the dcLNs was subsequently analyzed using ImageJ software, and normalized to the respective area.

### Drug Administration

*In vivo study*: Related drugs, doses, timing, and modes of administration were shown in Table S1 (Supporting Information). Before intracisternal infusion, the experimental animals received a single injection of the drugs dissolved in 0.1 ml normal saline. Animal control received 0.1 ml of normal saline.

*In vitro study*: Related drugs, doses, and timing were shown in Table S2 (Supporting Information). The cells were pre‐incubated with the drugs for 30 min prior to VLIUS treatment.

### Free‐Floating Immunofluorescence Staining

For free‐floating immunofluorescence staining, the 250 µm coronal slices were cut on a vibratome (MicroSlicer™ DTK‐1000N, DSK) and then incubated in 2% PBST (2% Triton X‐100 in PBS) for 1 d to promote tissue permeability. Slices were washed three times with PBS and blocked with fresh blocking buffer (10% normal goat serum, 1% Triton‐X100, 2.5% DMSO, and 0.2% sodium azide) for 1 d on a rocker at 4 °C. The slices were incubated with primary antibody in SignalStain^®^ antibody diluent (Cell Signaling, Cat No. 8112, USA) on a rocker for 2 days at 4 °C, and then washed three times with washing buffer (3% NaCl and 0.2% Triton‐X100 in PBS) for 1 h at room temperature (RT). The slices were kept in washing buffer on a rocker at 4 °C overnight. The slices were incubated with secondary antibody on a rocker for 2 days at 4 °C, and then washed three times with washing buffer, and kept in washing buffer on a rocker at 4 °C. After washing with PBS (three times), clear samples with RapiClear® for 1 h to promote transparency and then mounted with fresh RapiClear®. The primary antibodies used were rabbit anti‐TRPV4 (Cat. PA5‐41066, 1:100; Thermo Fisher Scientific), mouse anti‐GFAP (Cat No. MA5‐12023, 1:500; Thermo Fisher Scientific), mouse anti‐AQP4 (Cat No. sc‐32739, 1:100; Santa Cruz Biotechnology, Dallas, TX, USA), rabbit anti‐NeuN (cat. No. PA5‐78639, 1:200; Thermo Fisher Scientific), rabbit anti‐ZO1 (Cat. No. 61–7300, 1:200; Thermo Fisher Scientific), mouse anti‐Occludin (Cat. No. 33–1500, 1:200; Thermo Fisher Scientific), and mouse anti‐Claudin 5 (Cat. No. 35–2500, 1:200; Thermo Fisher Scientific). The secondary antibodies used were Alexa Fluor 633‐conjugated goat anti‐mouse (Cat No. A‐21050, 1:200; Thermo Fisher Scientific) and Alexa Fluor 488‐conjugated goat anti‐rabbit antibodies (Cat No. A‐27034, 1:200; Thermo Fisher Scientific). Samples were imaged using a laser scanning confocal microscope (Carl Zeiss LSM880) and analyzed using ImageJ software.

To analyze astrocyte volume within the glia limitans, GFAP expression was further observed by laser scanning confocal microscopy at a 63X objective to record fluorescence images of the glia limitans. These were acquired in 70–100 µm z‐stacks (≈0.69 µm z‐steps). The cell volume was further evaluated and analyzed using the Imaris software (Oxford Instruments, 9.5 version, UK). Z‐stack images were opened in Imaris in their native format. Z‐stacks were automatically reconstructed into a 3D model using the Imaris software without image preprocessing. A surface creation wizard was used to analyze the volume of each intact cell (with the same threshold).

### Cell Culture

C6 glioma cells (Bioresource Collection and Research Center, Hsinchu City, Taiwan), an immortalized rat glial cell line, were maintained in Dulbecco's modified Eagle's medium containing high glucose (DMEM) (Cat No. 12 100 046, Thermo Fisher Scientific) supplemented with 10% fetal bovine serum (Thermo Fisher Scientific) and a 1 × antibiotic–antimycotic (Cat No. 15 240 112, Thermo Fisher Scientific) at 37 °C in 5% CO_2_. Western blot analysis revealed that C6 cells natively expressed TRPV4 and AQP4 (data not shown). In addition, C6 cells were thought to possess astrocytic properties.^[^ ^50^ ^]^ As such, C6 cells were selected for subsequent in vitro studies.

### Live Cell Calcium Signal Imaging

A micropipette‐guided ultrasound system was used for the calcium influx assay.^[^ ^21^ ^]^ Cells (3 × 10^5^) were seeded on a 30 mm circular coverslip 18 h prior to the experiments. The cells were then washed twice with Hank's Balanced Salt Solution (HBSS) (Cat No. 14 025 134, Thermo Fisher Scientific) and incubated with 5 µM Fluo‐8 AM (Cat No. ab142773, Abcam, Bristol, UK) for 30 min. Next, the cells were washed thrice with HBSS and incubated in serum‐free DMEM (no phenol red, Cat No. 21 063 029, Thermo Fisher Scientific) for 30 min at RT. The coverslip was mounted on a microscopy chamber and placed under an Olympus IX71 microscope, and an ultrasound micropipette was placed near the targeted cells. Calcium influx images were recorded using the same exposure protocol: 10 s pretreatment followed by 3 s ultrasound stimulation and then a 57 s wait time. Stack images were analyzed using ImageJ software. The areas of interest were determined for the stacks, and a graph of the fluorescence intensity against time was plotted.

### Cell Surface Biotinylation

C6 glioma cells were plated in 24 well plates 1 d prior to each experiment. Cell surface proteins were biotinylated using the EZ‐Link^TM^ Sulfo‐NHS‐SS‐Biotin reagent (Cat No. 21 331, Thermo Fisher Scientific). Cells were then treated in three experimental conditions (VLIUS, agonist, antagonist, or inhibitor) and then incubated in 250 µL of 0.5 mg ml^−1^ biotinylation reagent in PBS on ice for 30 min. The unlabeled reagent was quenched with 25 mM glycine in PBS per well for 3–5 min. Cells were lysed in 100 µL Pierce^TM^ RIPA Buffer (Cat No. 89 900, Thermo Fisher Scientific) supplemented with protease inhibitor cocktail set III (Cat No. 539134‐1 set, 1:100, Merck Millipore, Rahway, NJ). The lysate was centrifuged at 16 000 g at 4 °C for 10 min to remove insoluble samples. Each lysate was analyzed using Coomassie protein assay reagent (Cat No. 1 856 209, Thermo Fisher Scientific) for normalization. Biotinylated proteins were immobilized out by incubation in 96‐well high‐sensitivity streptavidin microplates (Cat No. 6523–5, BioVision, Waltham, MA, USA) for 2 h at 4 °C with shaking. Plates were then blocked with 3% w/v bovine serum albumin (BSA) in PBS for 1 h at RT with shaking and then incubated on a shaker overnight at 4 °C with the anti‐AQP4 antibody (Cat No. GTX133151; GeneTex, San Antonio, TX, USA) diluted 1:400 in 0.1% PBST 20. Plates were washed three times with 0.1% PBST and incubated at RT for 1 h with horseradish peroxidase‐conjugated secondary antibody (Cat No. NA934, Merck Millipore) diluted 1:1000 in 0.05% PBST. Plates were washed with 0.1% PBST five times then once with PBS, and incubated with 3,3′,5,5′‐tetramethylbenzidine substrate solution (Cat No. N301, Thermo Fisher Scientific) for 20 min (protected from light). Absorbance was finally measured at 450 nm using a microplate reader (Infinite M200).

### AQP4 Surface Membrane Localization by Flow Cytometry Analysis

One hour following VLIUS stimulation, C6 cells were washed with cold PBS and resuspended in Accutase® cell detachment solution (Cat No. AT104, Innovative Cell Technologies, San Diego, CA, USA). Cells were fixed with 4% PFA for 15 min at RT, and then washed three times with PBS. As it was only detected AQP4 protein on the cell surface, the cells were not permeabilized (such as with Triton X‐100 or methanol treatment), as this facilitated antibody entry into the cells to detect intracellular AQP4 protein. Cells were blocked with blocking solution (1% w/v BSA in PBS) for 1 h at RT, and then incubated with the anti‐AQP4 antibody (GeneTex, Cat No. GTX133151, diluted 1:200 in block solution) overnight at 4 °C. The cells were washed three times with blocking solution and incubated at RT for 2 h with a secondary antibody (Cat No. A‐21428, Thermo Fisher Scientific) was diluted to 1:400 in a block solution. Cells were washed three times with PBS and analyzed using LSRII flow cytometry (BD Biosciences, East Rutherford, NJ, USA) at the flow cytometric analysis and sorting core facility at the National Taiwan University Hospital.

### AQP4 Surface Membrane Localization by Immunofluorescence Staining

Cells were seeded at 5 × 10^4^ cells on a 15 mm circular coverslip (in 24 well plates) 18 h prior to the experiments. After 1 h of VLIUS stimulation, C6 cells were washed with cold PBS and fixed by incubation in 4% PFA for 15 min at RT. Similarly, the cells were not permeabilized and only cell surface AQP4s were detected using the antibody. Cells were blocked with 1% w/v BSA in 0.1% PBS‐Tween 20 for 1 h at RT and then incubated with an anti‐AQP4 antibody (Cat No. GTX133151, GeneTex) diluted 1:400 in SignalStain^®^ antibody diluent (Cat No. 8112, Cell Signaling Technology, Danvers, MA, USA) overnight at 4 °C. Cells were washed three times with the blocking solution before being incubated at RT for 2 h with a secondary antibody (Thermo Fisher Scientific, Cat No. A‐21428; 1:200 dilution in SignalStain^®^ antibody). The cells were then counterstained with WGA and Alexa Fluor 488 conjugate (Cat No. W11261, Thermo Fisher Scientific) to determine the cell range. Finally, the cells were mounted using the EverBrite mounting medium with 4′,6‐diamidino‐2‐phenylindole (Cat No. 23 002, Biotium, Fremont, CA, USA). Images were recorded using an Olympus IX51 microscope with a ToupTek camera and with the same exposure parameters (Alexa Fluor™ 555: exposure time = 30 ms, gain = 200%; WGA‐Alexa Fluor™ 488: exposure time = 10 ms, gain = 200%). Images were analyzed using the ImageJ software.

### Statistical Analyses

All numerical data were expressed as the mean ± standard deviation and were analyzed using GraphPad Prism software (GraphPad Software, La Jolla, CA, USA). The Shapiro‐Wilk test was applied to test for normality. Statistical significance was assessed using the independent t‐test or its nonparametric equivalent, the Mann‐Whitney U test, for comparisons between two groups. One‐way analysis of variance (ANOVA) followed by Tukey's post‐hoc test, or the Kruskal‐Wallis test followed by Dunn's post‐hoc test, were used for multiple group comparisons. Statistical significance was set at *p* < 0.05. The statistical analyses and sample sizes used for each experiment were specified in the figure legends.

## Conflict of Interest

The authors declare no conflict of interest.

## Author Contributions

C.‐H.W. and W.‐H.L. contributed equally to this work and are co‐first authors. C.‐H.W., W.‐H.L. and W.‐S.C. conceived the study and designed the experiments. C.‐H.W., W.‐H.L., Y.‐C.C., M.‐Y.H. and Y.K. performed the experiments, developed the methods and analyzed the data. Y.‐C.C. and J.‐L.W. provided in vivo and in vitro ultrasound stimulation systems and conducted output calibration. C.‐H.W. and W.‐H.L. drafted the manuscript. J.‐L.W and W.‐S.C. supervised the study, guided the discussion, reviewed and edited the manuscript.

## Supporting information



Supporting Information

Supplemental Video 1

Supplemental Video 2

## Data Availability

The data that support the findings of this study are available from the corresponding author upon reasonable request.

## References

[1] a) J. J. Iliff , M. Wang , Y. Liao , B. A. Plogg , W. Peng , G. A. Gundersen , H. Benveniste , G. E. Vates , R. Deane , S. A. Goldman , E. A. Nagelhus , M. Nedergaard , Sci. Transl. Med. 2012, 4, 147ra111; 10.1126/scitranslmed.3003748 PMC3551275 22896675

[2] a) W. Peng , T. M. Achariyar , B. Li , Y. Liao , H. Mestre , E. Hitomi , S. Regan , T. Kasper , S. Peng , F. Ding , H. Benveniste , M. Nedergaard , R. Deane , Neurobiol. Dis. 2016, 93, 215; 27234656 10.1016/j.nbd.2016.05.015 PMC4980916

[3] a) J. J. Iliff , M. J. Chen , B. A. Plog , D. M. Zeppenfeld , M. Soltero , L. Yang , I. Singh , R. Deane , M. Nedergaard , J. Neurosci. 2014, 34, 16180; 25471560 10.1523/JNEUROSCI.3020-14.2014 PMC4252540

[4] a) H. Mestre , T. Du , A. M. Sweeney , G. Liu , A. J. Samson , W. Peng , K. N. Mortensen , F. F. Staeger , P. A. R. Bork , L. Bashford , E. R. Toro , J. Tithof , D. H. Kelley , J. H. Thomas , P. G. Hjorth , E. A. Martens , R. I. Mehta , O. Solis , P. Blinder , D. Kleinfeld , H. Hirase , Y. Mori , M. Nedergaard , Science. 2020, 367, aax7171; 10.1126/science.aax7171 PMC7375109 32001524

[5] A. J. Schain , A. Melo‐Carrillo , A. M. Strassman , R. Burstein , J. Neurosci. 2017, 37, 2904. 28193695 10.1523/JNEUROSCI.3390-16.2017 PMC5354333

[6] A. Carotenuto , L. Cacciaguerra , E. Pagani , P. Preziosa , M. Filippi , M. A. Rocca , Brain. 2021, 145, 2785. 10.1093/brain/awab454 34919648

[7] A. Zamani , A. K. Walker , B. Rollo , K. L. Ayers , R. Farah , T. J. O'Brien , D. K. Wright , Transl. Neurodegener. 2022, 11, 17. 35287738 10.1186/s40035-022-00291-4 PMC8922788

[8] T. M. Achariyar , B. Li , W. Peng , P. B. Verghese , Y. Shi , E. McConnell , A. Benraiss , T. Kasper , W. Song , T. Takano , D. M. Holtzman , M. Nedergaard , R. Deane , Mol. Neurodegener. 2016, 11, 74. 27931262 10.1186/s13024-016-0138-8 PMC5146863

[9] H. Lee , L. Xie , M. Yu , H. Kang , T. Feng , R. Deane , J. Logan , M. Nedergaard , H. Benveniste , J. Neurosci. 2015, 35, 11034. 26245965 10.1523/JNEUROSCI.1625-15.2015 PMC4524974

[10] H. Mestre , J. Tithof , T. Du , W. Song , W. Peng , A. M. Sweeney , G. Olveda , J. H. Thomas , M. Nedergaard , D. H. Kelley , Nat. Commun. 2018, 9, 4878. 30451853 10.1038/s41467-018-07318-3 PMC6242982

[11] B. T. Kress , J. J. Iliff , M. Xia , M. Wang , H. S. Wei , D. Zeppenfeld , L. Xie , H. Kang , Q. Xu , J. A. Liew , B. A. Plog , F. Ding , R. Deane , M. Nedergaard , Ann. Neurol. 2014, 76, 845. 25204284 10.1002/ana.24271 PMC4245362

[12] a) C. Gakuba , T. Gaberel , S. Goursaud , J. Bourges , C. Di Palma , A. Quenault , S. Martinez de Lizarrondo , D. Vivien , M. Gauberti , Theranostics. 2018, 8, 710; 29344300 10.7150/thno.19154 PMC5771087

[13] H. Mestre , L. M. Hablitz , A. L. Xavier , W. Feng , W. Zou , T. Pu , H. Monai , G. Murlidharan , R. M. Castellanos Rivera , M. J. Simon , M. M. Pike , V. Pla , T. Du , B. T. Kress , X. Wang , B. A. Plog , A. S. Thrane , I. Lundgaard , Y. Abe , M. Yasui , J. H. Thomas , M. Xiao , H. Hirase , A. Asokan , J. J. Iliff , M. Nedergaard , Elife. 2018, 7, 40070. 10.7554/eLife.40070 PMC6307855 30561329

[14] P. Kitchen , M. M. Salman , A. M. Halsey , C. Clarke‐Bland , J. A. MacDonald , H. Ishida , H. J. Vogel , S. Almutiri , A. Logan , S. Kreida , T. Al‐Jubair , J. Winkel Missel , P. Gourdon , S. Tornroth‐Horsefield , M. T. Conner , Z. Ahmed , A. C. Conner , R. M. Bill , Cell. 2020, 181, 784. 32413299 10.1016/j.cell.2020.03.037 PMC7242911

[15] V. Benfenati , M. Caprini , M. Dovizio , M. N. Mylonakou , S. Ferroni , O. P. Ottersen , M. Amiry‐Moghaddam , Proc. Natl. Acad. Sci. U S A. 2011, 108, 2563. 21262839 10.1073/pnas.1012867108 PMC3038710

[16] M. M. Maneshi , B. Maki , R. Gnanasambandam , S. Belin , G. K. Popescu , F. Sachs , S. Z. Hua , Sci. Rep. 2017, 7, 39610. 28045032 10.1038/srep39610 PMC5206744

[17] W.‐H. Liao , M.‐Y. Hsiao , Y. Kung , H.‐L. Liu , J.‐C. Béra , C. Inserra , W.‐S. Chen , J. Adv. Res. 2020, 26, 15. 33133680 10.1016/j.jare.2020.06.012 PMC7584681

[18] M. Aryal , M. M. Azadian , A. R. Hart , N. Macedo , Q. Zhou , E. L. Rosenthal , R. D. Airan , J. Control Release. 2022, 349, 434. 35798095 10.1016/j.jconrel.2022.06.067

[19] Y. Lee , Y. Choi , E. J. Park , S. Kwon , H. Kim , J. Y. Lee , D. S. Lee , Sci. Rep. 2020, 10, 16144. 32999351 10.1038/s41598-020-73151-8 PMC7527457

[20] J. Lim , H. H. Tai , W. H. Liao , Y. C. Chu , C. M. Hao , Y. C. Huang , C. H. Lee , S. S. Lin , S. Hsu , Y. C. Chien , D. M. Lai , W. S. Chen , C. C. Chen , J. L. Wang , Elife. 2021, 10, 61660. 10.7554/eLife.61660 PMC8510583 34569932

[21] Y. C. Chu , J. Lim , C. H. Lai , M. C. Tseng , Y. S. Chu , J. L. Wang , Ultrasound Med. Biol. 2021, 47, 1775. 33931285 10.1016/j.ultrasmedbio.2021.03.020

[22] a) O. Butenko , D. Dzamba , J. Benesova , P. Honsa , V. Benfenati , V. Rusnakova , S. Ferroni , M. Anderova , PLoS One. 2012, 7, e39959; 22761937 10.1371/journal.pone.0039959 PMC3384594

[23] a) K. M. Dunn , D. C. Hill‐Eubanks , W. B. Liedtke , M. T. Nelson , Proc. Natl. Acad. Sci. U S A. 2013, 110, 6157; 23530219 10.1073/pnas.1216514110 PMC3625327

[24] J. J. Iliff , M. Wang , D. M. Zeppenfeld , A. Venkataraman , B. A. Plog , Y. Liao , R. Deane , M. Nedergaard , J. Neurosci. 2013, 33, 18190. 24227727 10.1523/JNEUROSCI.1592-13.2013 PMC3866416

[25] P. Rajasekhar , D. P. Poole , N. A. Veldhuis , Adv. Pharmacol. 2017, 79, 117. 28528666 10.1016/bs.apha.2017.03.002

[26] H. Mestre , Y. Mori , M. Nedergaard , Trends Neurosci. 2020, 43, 458. 32423764 10.1016/j.tins.2020.04.003 PMC7331945

[27] A. O. Jo , D. A. Ryskamp , T. T. Phuong , A. S. Verkman , O. Yarishkin , N. MacAulay , D. Krizaj , J. Neurosci. 2015, 35, 13525. 26424896 10.1523/JNEUROSCI.1987-15.2015 PMC4588615

[28] a) Y. Takayama , K. Shibasaki , Y. Suzuki , A. Yamanaka , M. Tominaga , FASEB J. 2014, 28, 2238; 24509911 10.1096/fj.13-243436

[29] a) A. Iuso , D. Krizaj , Channels (Austin). 2016, 10, 172; 26760501 10.1080/19336950.2016.1140956 PMC4954570

[30] M. M. Salman , P. Kitchen , M. N. Woodroofe , J. E. Brown , R. M. Bill , A. C. Conner , M. T. Conner , Eur. J. Neurosci. 2017, 46, 2542. 28925524 10.1111/ejn.13723 PMC5765450

[31] a) J. Jorgacevski , R. Zorec , M. Potokar , Cells. 2020, 9, 2622; 33297299 10.3390/cells9122622 PMC7762321

[32] J. Liu , Y. Yang , X. Li , P. Zhang , Y. Qi , H. Hu , in (Ed.: M. Fukuda ), Academic Press, 2010.

[33] D. Becker , C. Blase , J. Bereiter‐Hahn , M. Jendrach , J. Cell Sci. 2005, 118, 2435. 15923656 10.1242/jcs.02372

[34] V. Benfenati , M. Amiry‐Moghaddam , M. Caprini , M. N. Mylonakou , C. Rapisarda , O. P. Ottersen , S. Ferroni , Neuroscience. 2007, 148, 876. 17719182 10.1016/j.neuroscience.2007.06.039

[35] T. J. Lohela , T. O. Lilius , M. Nedergaard , Nat. Rev. Drug Discov. 2022, 21, 763. 35948785 10.1038/s41573-022-00500-9

[36] a) H. L. Liu , P. H. Hsu , C. Y. Lin , C. W. Huang , W. Y. Chai , P. C. Chu , C. Y. Huang , P. Y. Chen , L. Y. Yang , J. S. Kuo , K. C. Wei , Radiology. 2016, 281, 99; 27192459 10.1148/radiol.2016152444

[37] S. Xu , D. Ye , L. Wan , Y. Shentu , Y. Yue , M. Wan , H. Chen , Ultrasound Med. Biol. 2019, 45, 2758. 31378549 10.1016/j.ultrasmedbio.2019.07.004

[38] D. Ye , S. Chen , Y. Liu , C. Weixel , Z. Hu , J. Yuan , H. Chen , Proc. Natl. Acad. Sci. U S A. 2023, 120, e2212933120. 37186852 10.1073/pnas.2212933120 PMC10214201

[39] D. G. Blackmore , F. Turpin , T. Palliyaguru , H. T. Evans , A. Chicoteau , W. Lee , M. Pelekanos , N. Nguyen , J. Song , R. K. P. Sullivan , P. Sah , P. F. Bartlett , J. Gotz , Mol. Psychiatry. 2021, 26, 6975. 34040151 10.1038/s41380-021-01129-7 PMC8760044

[40] S. S. Yoo , E. Kim , K. Kowsari , J. Van Reet , H. C. Kim , K. Yoon , Sci. Rep. 2023, 13, 12339. 37524783 10.1038/s41598-023-39640-2 PMC10390479

[41] J. A. Stokum , M. S. Kwon , S. K. Woo , O. Tsymbalyuk , R. Vennekens , V. Gerzanich , J. M. Simard , Glia. 2018, 66, 108. 28906027 10.1002/glia.23231 PMC5759053

[42] a) B. Reiter , R. Kraft , D. Gunzel , S. Zeissig , J. D. Schulzke , M. Fromm , C. Harteneck , FASEB J. 2006, 20, 1802; 16940152 10.1096/fj.06-5772com

[43] a) P. Jie , Z. Lu , Z. Hong , L. Li , L. Zhou , Y. Li , R. Zhou , Y. Zhou , Y. Du , L. Chen , L. Chen , Mol. Neurobiol. 2016, 53, 8; 25399955 10.1007/s12035-014-8992-2

[44] S. Holstein‐Ronsbo , Y. Gan , M. J. Giannetto , M. K. Rasmussen , B. Sigurdsson , F. R. M. Beinlich , L. Rose , V. Untiet , L. M. Hablitz , D. H. Kelley , M. Nedergaard , Nat. Neurosci. 2023, 26, 1042. 37264158 10.1038/s41593-023-01327-2 PMC10500159

[45] a) D. Guo , J. Zou , N. Rensing , M. Wong , PLoS One. 2017, 12, e0170005; 28107381 10.1371/journal.pone.0170005 PMC5249218

[46] V. Benfenati , G. P. Nicchia , M. Svelto , C. Rapisarda , A. Frigeri , S. Ferroni , J. Neurochem. 2007, 100, 87. 17064359 10.1111/j.1471-4159.2006.04164.x

[47] S. Baratchi , P. Keov , W. G. Darby , A. Lai , K. Khoshmanesh , P. Thurgood , P. Vahidi , K. Ejendal , P. McIntyre , Front. Pharmacol. 2019, 10, 6. 30728775 10.3389/fphar.2019.00006 PMC6351496

[48] A. Imura , Y. Tsuji , M. Murata , R. Maeda , K. Kubota , A. Iwano , C. Obuse , K. Togashi , M. Tominaga , N. Kita , K. Tomiyama , J. Iijima , Y. Nabeshima , M. Fujioka , R. Asato , S. Tanaka , K. Kojima , J. Ito , K. Nozaki , N. Hashimoto , T. Ito , T. Nishio , T. Uchiyama , T. Fujimori , Y. Nabeshima , Science. 2007, 316, 1615. 17569864 10.1126/science.1135901

[49] A. L. R. Xavier , N. L. Hauglund , S. von Holstein‐Rathlou , Q. Li , S. Sanggaard , N. Lou , I. Lundgaard , M. Nedergaard , J. Vis. Exp. 2018, 23, 57378. 10.3791/57378 PMC6101354 29889209

[50] F. Galland , M. Seady , J. Taday , S. S. Smaili , C. A. Goncalves , M. C. Leite , Neurochem. Int. 2019, 131, 104538. 31430518 10.1016/j.neuint.2019.104538

