# Supplementary material for: Very Low‐Intensity Ultrasound Facilitates Glymphatic Influx and Clearance via Modulation of the TRPV4‐AQP4 Pathway
Source: Adv Sci (Weinh). 2024 Nov 4;11(47):2401039. doi: 10.1002/advs.202401039 (PMC11653672; doi:10.1002/advs.202401039)
Supplement: Supplementary file 1 — Supporting Information [file ADVS-11-2401039-s002.docx]

**Supplemental Materials**


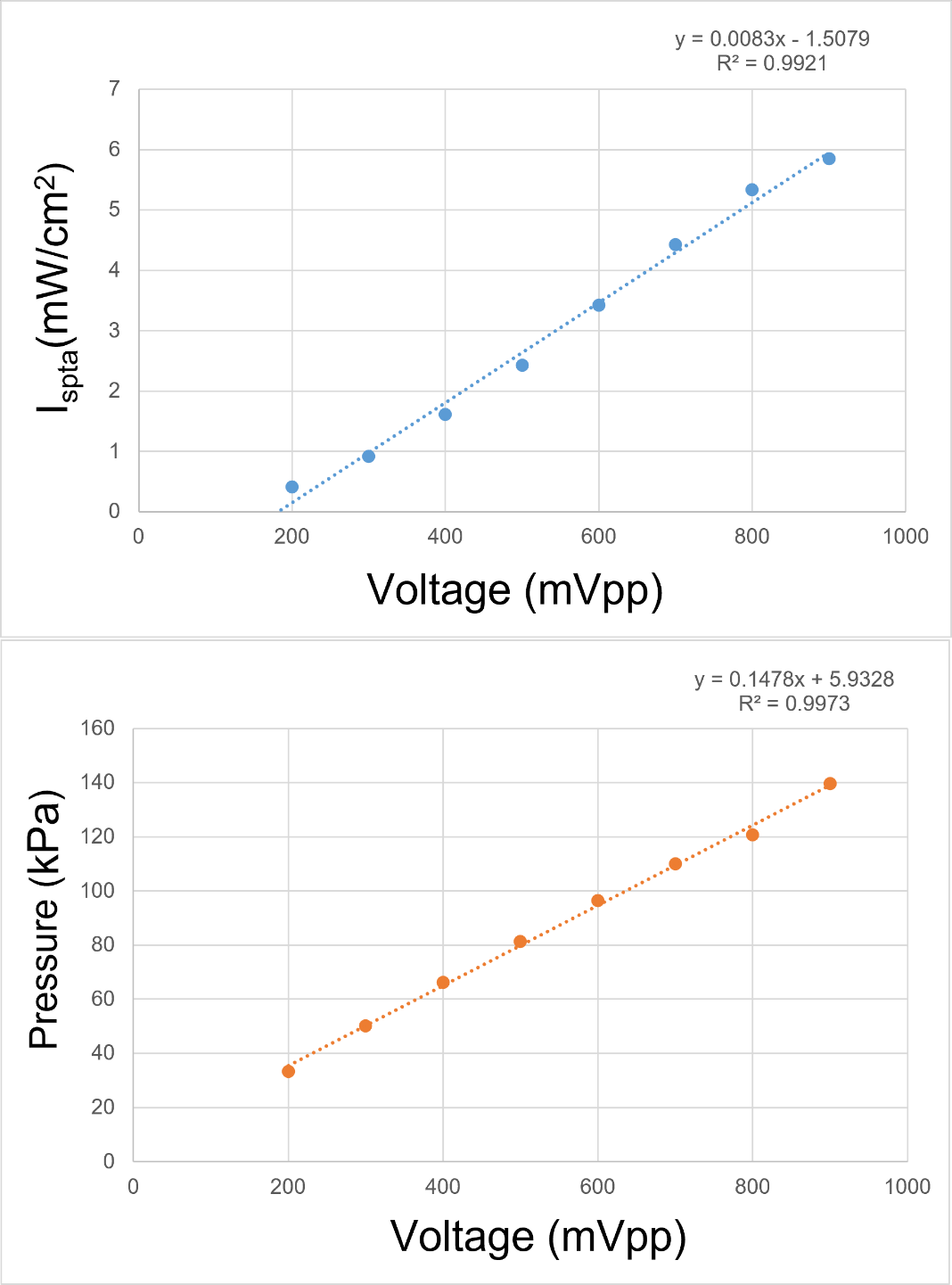


**Fig. S1:** Spatial-Peak Temporal Average Intensity (I_spta_) or acoustic pressure exhibits a linear relationship with input voltage.


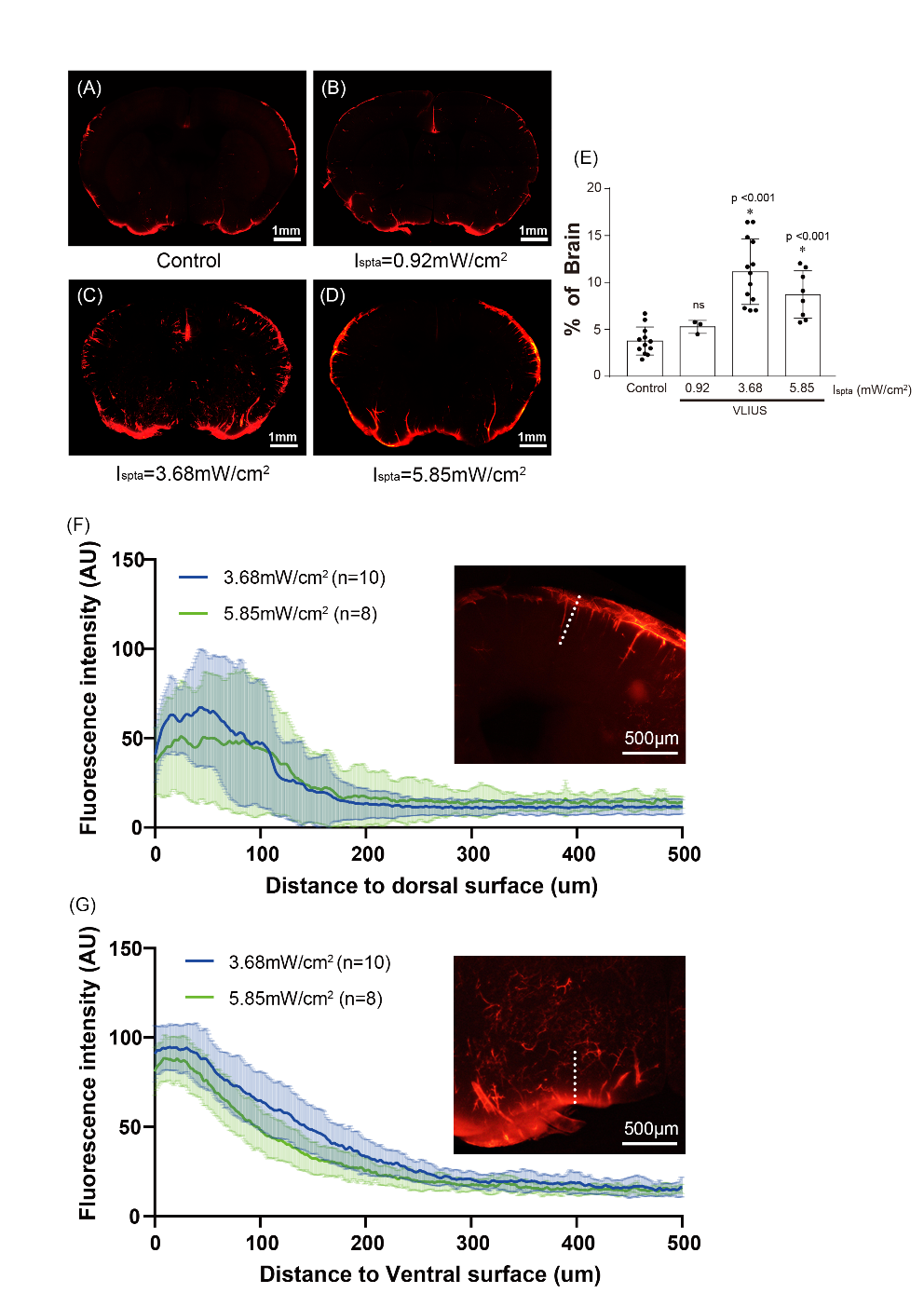


**Fig. S2:** Enhancement of glymphatic system function using ultrasound at different intensities **(A-E)**. Comparing the effects of two different intensities of VLIUS stimulation, 3.68 and 5.85 mW/cm^2^, on the tracer penetration depth from the dorsal **(F)** and ventral surfaces **(G)**. No significant differences were observed between the two. The results, for which the data are presented as the mean ± SD (error bars denote SD), shown in Figure S2(E) were analyzed using ANOVA followed by Tukey's post-hoc test to assess between-group differences. An asterisk indicates *p* < 0.05 in comparison with the control group. ns: non-significant.


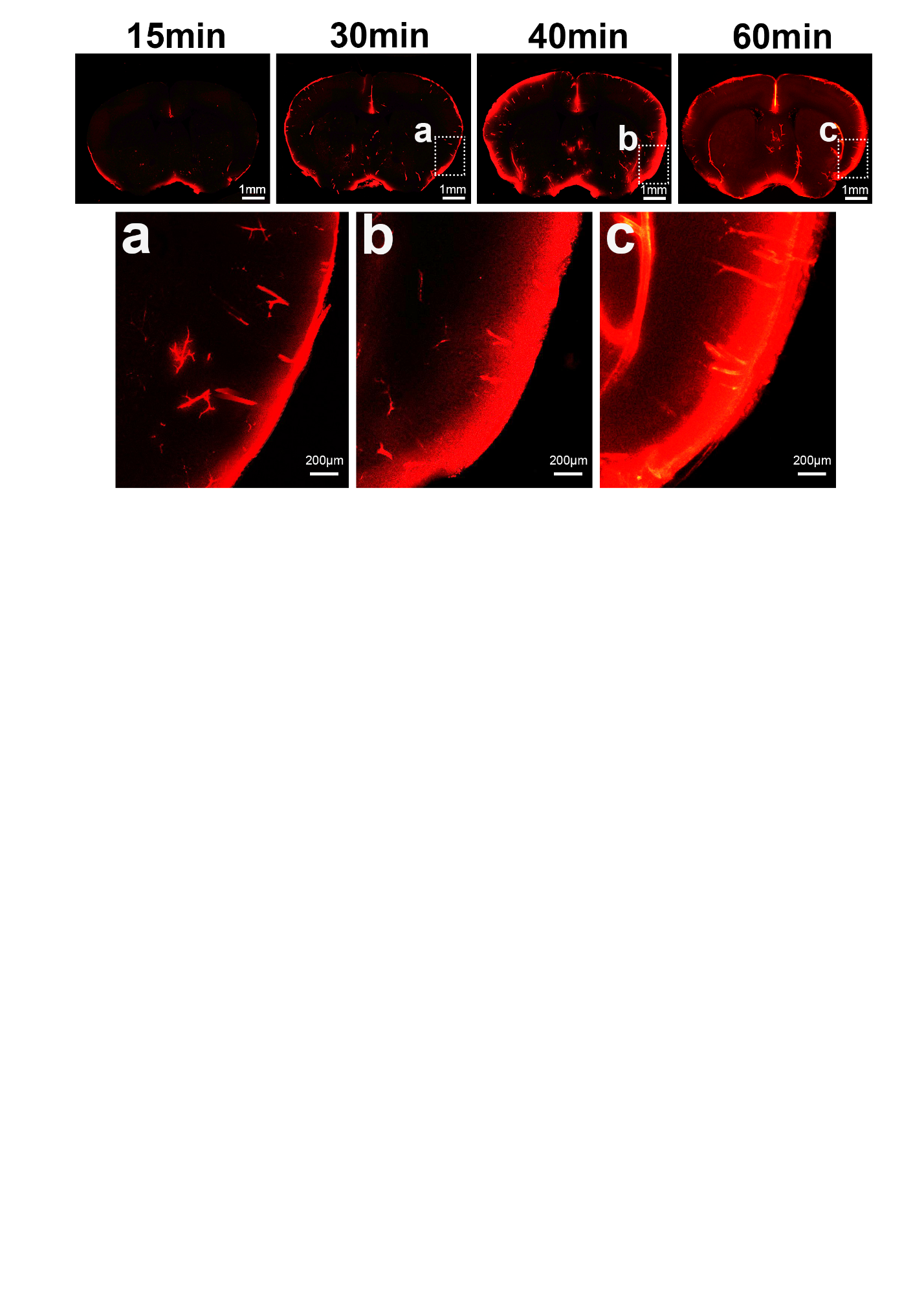


**Fig. S3:** Diffusion of the tracer from the brain surface to the brain parenchyma over time (without VLIUS). The unaltered real-time outputs of image capture combined with extended depth of field (EDF), without any additional post-processing or threshold adjustments, are shown. As the observation time was prolonged, a higher signal intensity was observed in the deeper brain parenchyma. This indicates that, following tracer cisterna magna injection, the tracer diffuses from the outer regions (subarachnoid space or paravascular space) into the parenchyma over time.


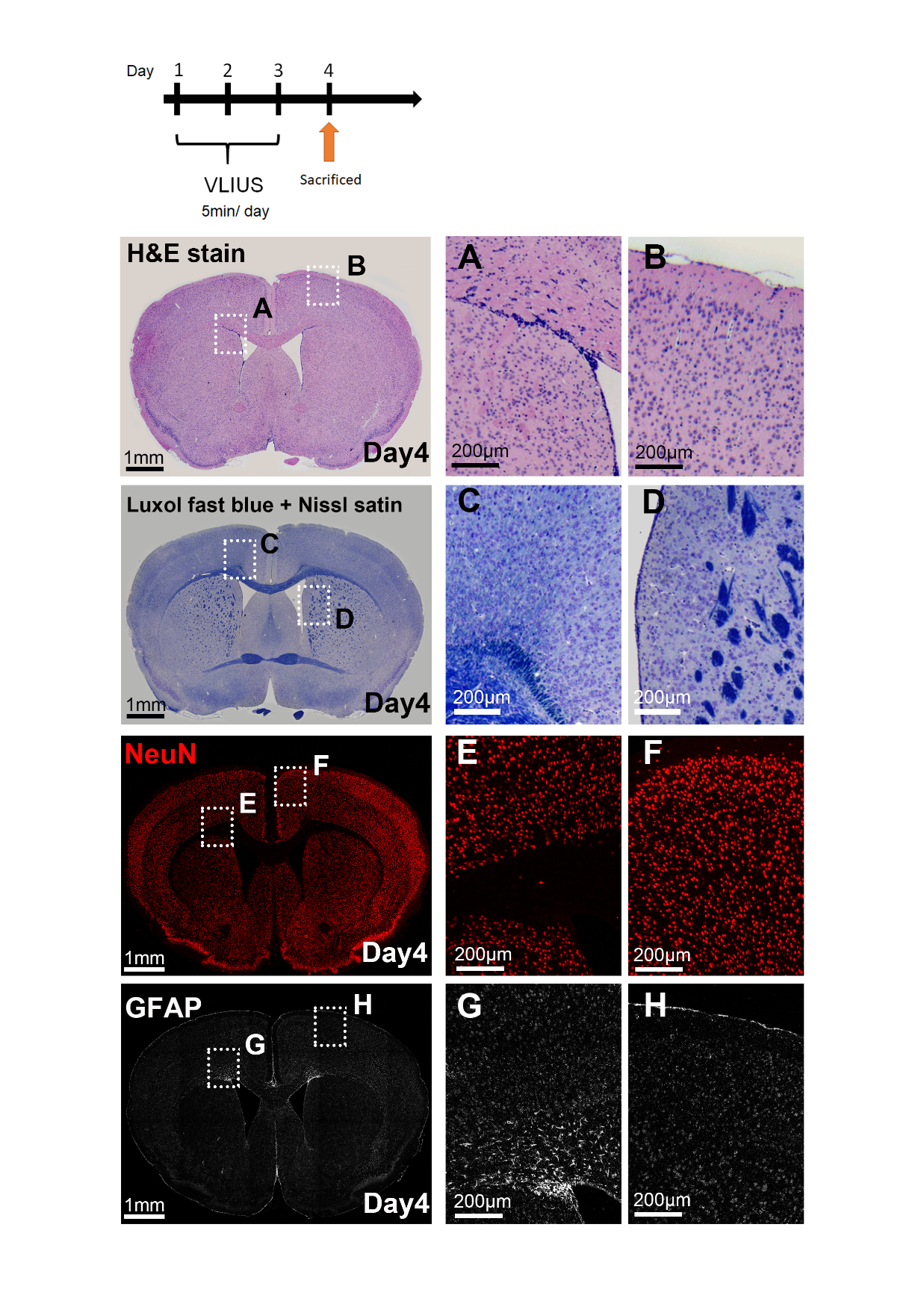


**Fig. S4:** Safety evaluation of VLIUS stimulation at day 4.
**(A, B)** Hematoxylin and Eosin staining showing no significant tissue changes. **(C, D)** Luxol fast blue and Nissl staining showing no significant myelin sheath changes or decrease in neuronal cells. **(E, F)** Neuronal nuclei staining showing no decrease in mature neurons. **(G, H)** GFAP staining showing no increase of astrocytes, indicating no neuroinflammation.


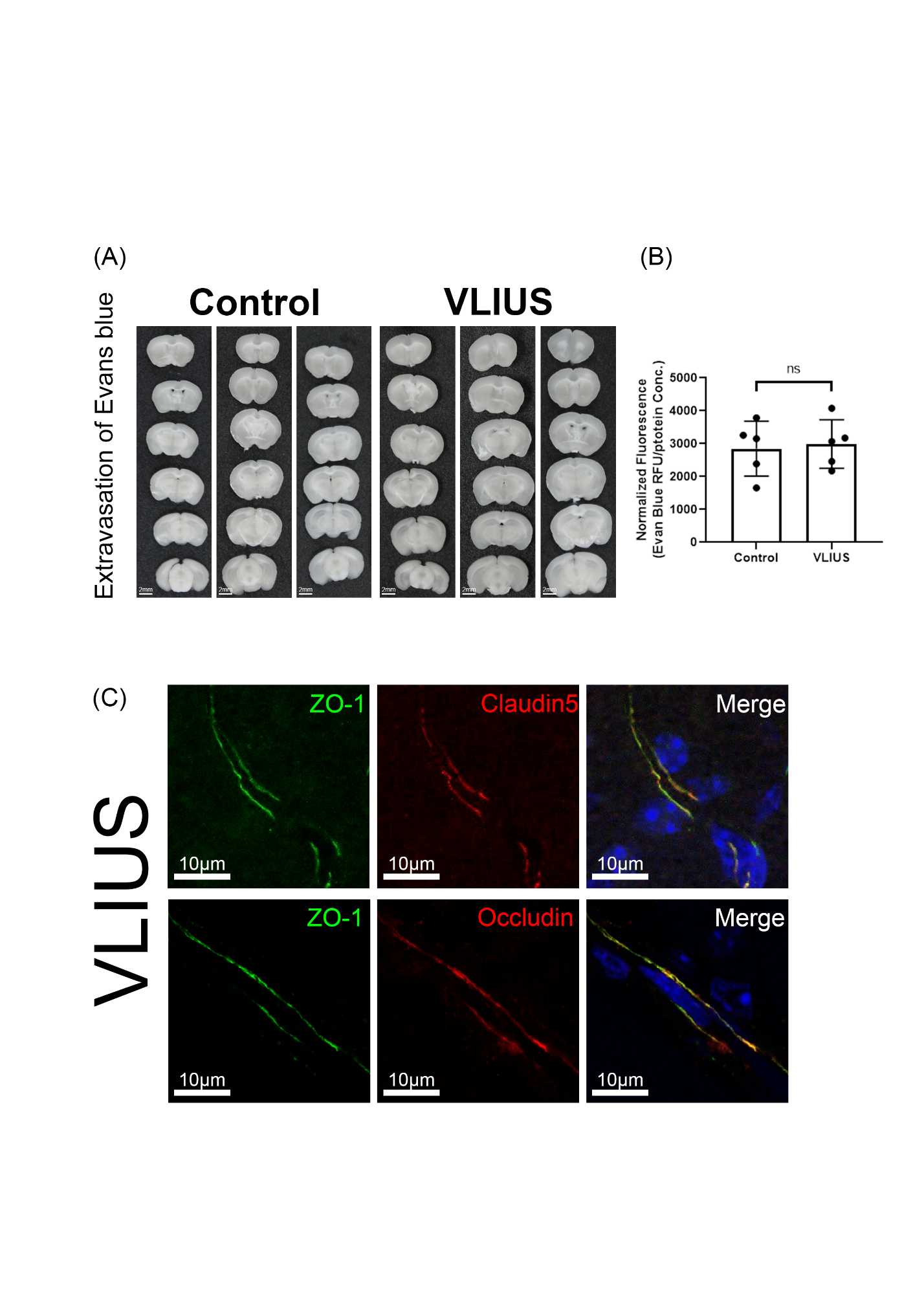


**Fig. S5:** Assessment of blood-brain barrier integrity following VLIUS stimulation.
**(A)** Evans blue extravasation analysis with quantification **(B)** showing that the BBB integrity was not disrupted by VLIUS stimulation. **(C)** The co-localization of ZO1-Claudin5 and ZO1-Occludin remains unaffected following VLIUS stimulation. The results, for which the data are presented as the mean ± SD (error bars denote SD), shown in Figure S5(B) were analyzed using an independent t-test to assess between-group differences. ns: non-significant.


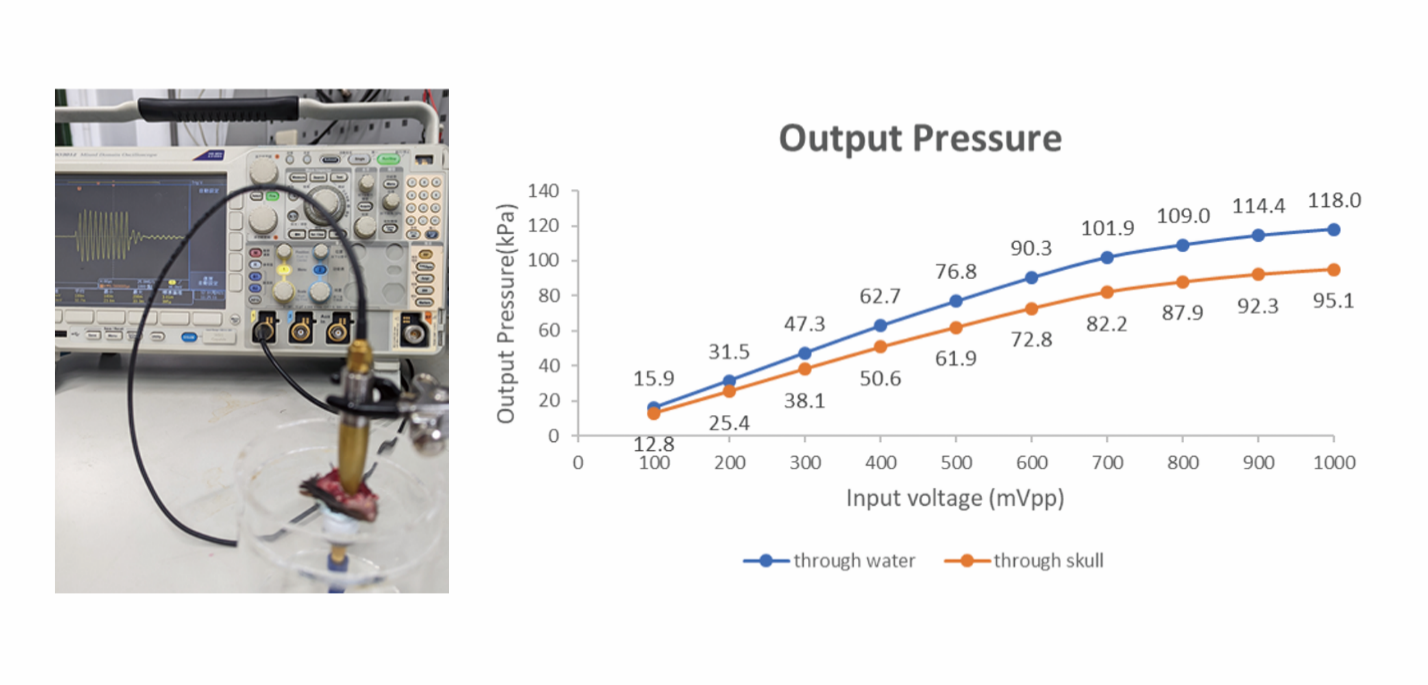


**Fig. S6:** Measurement of the pressure decay of VLIUS following transcranial transmission.
(Left) Experimental set-up of the VLIUS output pressure through the skull.
(Right) Relationship between the output pressure (kPa) and input voltage (mVpp). An approximately 20% decay of output pressure during transcranial propagation was observed in comparison with that through the water.

**Table S1.** Drug, dosage and administration for in vivo experiments

| **Function** | **Name** | **Dosage** | **Company (Cat. No)** | **Mode of administration** | **Drug administration before intracisternal infusion** |
| --- | --- | --- | --- | --- | --- |
| TRPV4 agonist | GSK1016790A | 5µg/kg | abcam (ab146191) | IV | Approximately 15 min |
| TRPV4 antagonist | GSK2193874 | 2mg/kg | MCE (HY-100720) | IV | Approximately 15 min |
| TRPV4 antagonist | HC067047 | 2mg/kg | abcam(ab145868) | IV | Approximately 15 min |
| AQP4 inhibitor | AER271 | 10mg/kg | MOLNOVA (M15401) | IP | Approximately 25 min |
| AQP4 inhibitor | TGN020 | 100mg/kg | MOLNOVA (M19928) | IP | Approximately 25 min |
| Calmodulin inhibitor | Trifluoperazine | 10mg/kg | TargetMol (T1222) | SC | More than 30 min |

AQP4, aquaporin-4; IV, intravenous injection; IP, intraperitoneal injection; SC, subcutaneous injection; TRPV4, transient receptor potential vanilloid-4.

**Table S2.** Drug and dosage for in vitro experiments

| **Function** | **Name** | **Concentration** |
| --- | --- | --- |
| TRPV4 agonist | GSK1016790A | 2µM |
| TRPV4 antagonist | GSK2193874 | 100nM |
| Calmodulin inhibitor | Trifluoperazine | 20.8µM |

TRPV4, transient receptor potential vanilloid-4
